# Supplementary material for: Solar reduction of carbon dioxide on copper-tin electrocatalysts with energy conversion efficiency near 20%
Source: Nat Commun. 2022 Oct 6;13:5898. doi: 10.1038/s41467-022-33049-7 (PMC9537560; doi:10.1038/s41467-022-33049-7)
Supplement: Supplementary file 1 — Supplementary Information File [file 41467_2022_33049_MOESM1_ESM.pdf]

Supplementary information for

Solar reduction of carbon dioxide on copper-tin electrocatalysts with energy conversion efficiency near 20%

Jing Gao[a*#], Jun Li[a#], Yuhang Liu[a#], Meng Xia[a], Y. Zou Finfrock[b], Shaik M. Zakeeruddin[a], Dan Ren[c,a*] and Michael Grätzel[a*]

a Laboratory of Photonics and Interfaces, École Polytechnique Fédérale de Lausanne, 1015 Lausanne, Switzerland

b. Structural Biology Center, X-ray Science Division, Argonne National Laboratory, Lemont, IL 60439, USA

c. Current affiliation: School of Chemical Engineering and Technology, Xi'an Jiaotong University, 710049 Xi'an, China

*Correspondence should be addressed to jing.gao@epfl.ch, dan.ren@xjtu.edu.cn and michael.graetzel@epfl.ch

S1 Supplementary Notes 2

Note 1. Calculation of Gibbs free energy 2

Note 2. Calculation of faradaic efficiency 3

Note 3. Calculation of solar-to-fuel (STF) conversion efficiency 4

Note 4. Calculation of CO production rate 5

S2 Supplementary Figures 6

S2.1 Additional characterization data for as-prepared samples 6

S2.2 Additional characterization data for electrocatalysts after reduction 13

S2.3 Additional electrochemistry data for Cu and Cu-Sn catalysts in 0.5 M $KHCO_3$ 22

S2.4 Electrocatalytic performance of Cu-Sn catalysts in 1 M KOH 24

S2.5 Morphological and electrochemical characterization of planar CuO supported SnO_2 catalyst 25

S2.6 Current against geometric surface area and electrochemical active surface area 27

S2.7 Effect of thickness of sputtered Sn and ALD-SnO_2 on the catalytic performance and morphology 28

S2.8 Electrocatalytic performance of bare sputtered Sn and ALD-SnO_2 catalysts 31

S2.9 Additional data for stability test 32

S2.10 Additional data for X-ray absorption spectroscopy 34

S2.11 Additional data for CO adsorption 36

S2.12 Additional data for in situ Raman spectroscopy 41

S2.13 Additional data for solar-driven CO_2 reduction system 44

S3 Supplementary Tables 50

S4 Supplementary References 61

S1 Supplementary Notes

Note 1. Calculation of Gibbs free energy

In order to determine whether the replacement reaction between sputtered Sn and CuO takes place spontaneously, here we calculate the change in Gibbs free energy of this reaction based on the Gibbs free energy equation:

$$Sn + 2CuO \rightleftharpoons SnO_2 + Cu$$

$$\Delta G = \Delta H - T\Delta S = -261\ kJ\ mol^{-1}$$

where ΔG, ΔH and ΔS are the changes of Gibbs free energy, enthalpy and entropy of the reaction, T represents room temperature (298 K). From the standard thermodynamic data of the two molecules and elements provided in the NIST WebBook[1], the ΔG of above reaction is calculated as -261 kJ mol^{-1}.

Note 2. Calculation of faradaic efficiency

The faradaic efficiency for each product X was calculated by the following equation:

$$FE(X) = \frac{electrons\ used\ for\ producing\ X}{total\ electrons\ for\ electrolysis} \times 100\%$$

For gaseous products, the faradaic efficiency was calculated as the average of last three injections (totally four injections) at each current density, since the gas phase requires 10-20 minutes for reaching equilibrium. The liquid products that dissolved in electrolyte were collected after each electrolysis and quantified by HPLC. For each current density, three measurements were averaged using fresh catalyst and electrolyte.

The partial current density for each product X is defined as:

$$j(X) = j_{total} \times FE(X)$$

Note 3. Calculation of solar-to-fuel (STF) conversion efficiency

In PV-EC CO_2 reduction system, the STF conversion efficiency is defined as the percentage of chemical energy stored against the illuminated solar energy. The efficiency is the multiplier of solar-to-electricity (STE) and electricity-to-fuel (ETF), where STE and ETF in the integrated system are respectively determined as:

$$\eta_{STE} = \frac{electrical\ power_{output}}{illumiantion\ power_{input}} = \frac{j_{op} \times V_{op}}{P_{input}}$$

$$\eta_{ETF} = \frac{chemical\ energy}{electrical\ energy} = \frac{j_{op} \times FE_{fuel} \times E_{fuel} \times \Delta t}{j_{op} \times V_{op} \times \Delta t}$$

Where j_{op} and V_{op} are the current density and voltage under the operating condition (note that here j_{op} is normalized to the working area of solar cell), P_{input} is the illumination power of incident light (here is 100 mA cm^{-2}), E_{fuel} is the thermodynamic potential of the overall reaction for specific product, FE_{fuel} is the faradaic efficiency of specific product. Thus, STF conversion efficiency can be calculated as:

$$\eta_{STF} = \eta_{STE} \times \eta_{ETF} = \frac{j_{op} \times V_{op}}{P_{input}} \times \frac{FE_{CO} \times E^{o}_{CO/CO2}}{V_{cell}} = \frac{j_{op} E_{fuel} FE_{fuel}}{P_{input}}$$

Here we take a calculation of average efficiency of solar-to-CO conversion as an example. The average current density of unassisted solar-driven CO_2 reduction is 14.8 mA cm^{-2}, the thermodynamic potential of the overall reaction for CO is 1.34 V, and the average FE of CO during 2-hr electrolysis is 98.9%. The solar-to-CO conversion efficiency is calculated as:

$$\eta_{solar-to-CO} = \frac{14.8\ mA\ cm^{-2} \times 1.34\ V\ \times 98.9\%}{100\ mW\ cm^{-2}} = 19.61\%$$

Note 4. Calculation of CO production rate

The production rate of CO during solar-driven CO_2 reduction PR(CO) in terms of mg h^{-1} cm^{-2} is calculated by normalizing the photocurrent to geometric surface area of CuO-$SnO_{2\,ALD}$ catalyst (0.25 cm^2). Here we take a calculation of average production rate of CO as an example. The average photocurrent density and faradaic efficacy of CO are 54.5 mA cm^{-2} and 98.5%, respectively. The PR(CO)is calculated as:

$$\mathrm{PR(CO)} = \frac{j_{CO}}{n \times F} = \frac{54.5\ mA\ cm^{-2} \times 98.5\%}{2 \times 96485\ C\ mol^{-1}} = 28\ mg\ h^{-1}\ cm^{-2}$$

S2 Supplementary Figures

S2.1 Additional characterization data for as-prepared samples

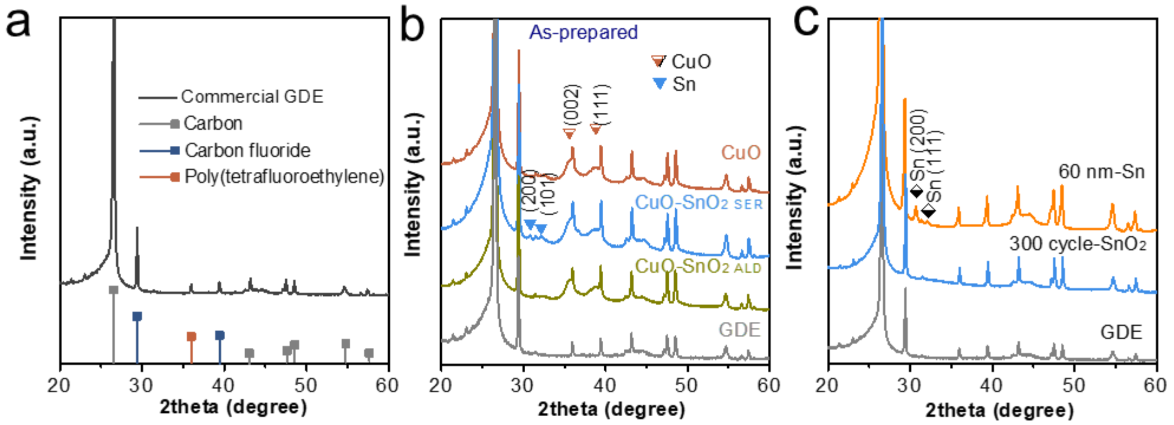

Supplementary Fig. 1. Structural characterizations of substrate and catalysts. (**a**) X-ray diffractogram of bare GDE (gas diffusion electrode) substrate (PDF #00-026-1076 for carbon, PDF #00-061-1414 for $(C_2F_4)_n$ and PDF #00-055-0072 for CF_4). (**b**) X-ray diffractograms of as-prepared CuO substrate (PDF #00-002-1040 for CuO), CuO-SnO_2 SER (PDF #01-086-2264 for Sn), CuO-SnO_2 ALD samples (PDF #00-002-1040 for CuO) and GDE substrate. (**c**) X-ray diffractograms of bare sputtered-Sn with a thickness of 60 nm and ALD-SnO_2 overlayer with deposition cycles of 300 (corresponding to a thickness of ~81 nm).

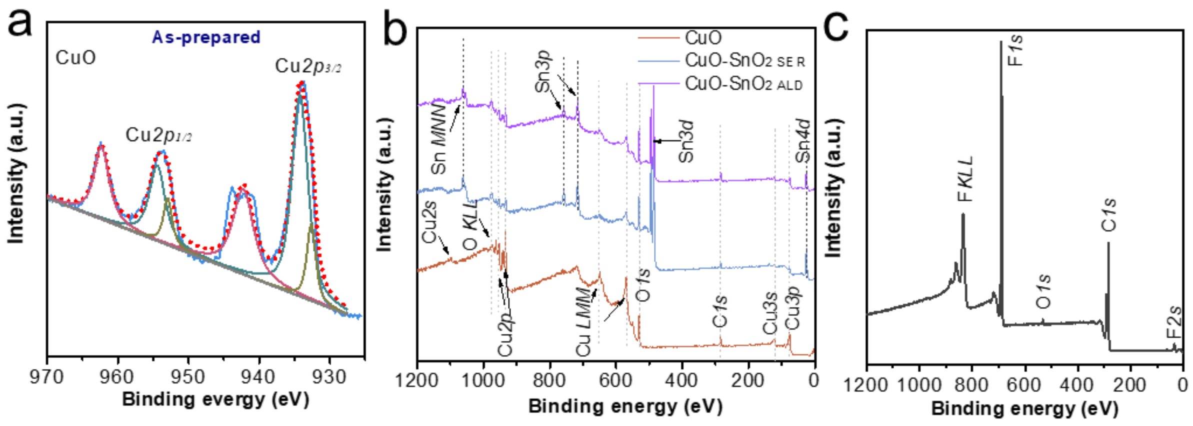

Supplementary Fig. 2. Surface chemical characterizations of substrate and catalysts. (**a**) high-resolution XPS spectrum of Cu *2p* of the as-prepared CuO sample. (**b**) XPS survey scan of the as-prepared CuO, CuO-SnO_2 SER and CuO-SnO_2 ALD samples. (**c**) XPS survey scan of bare GDE substrate.

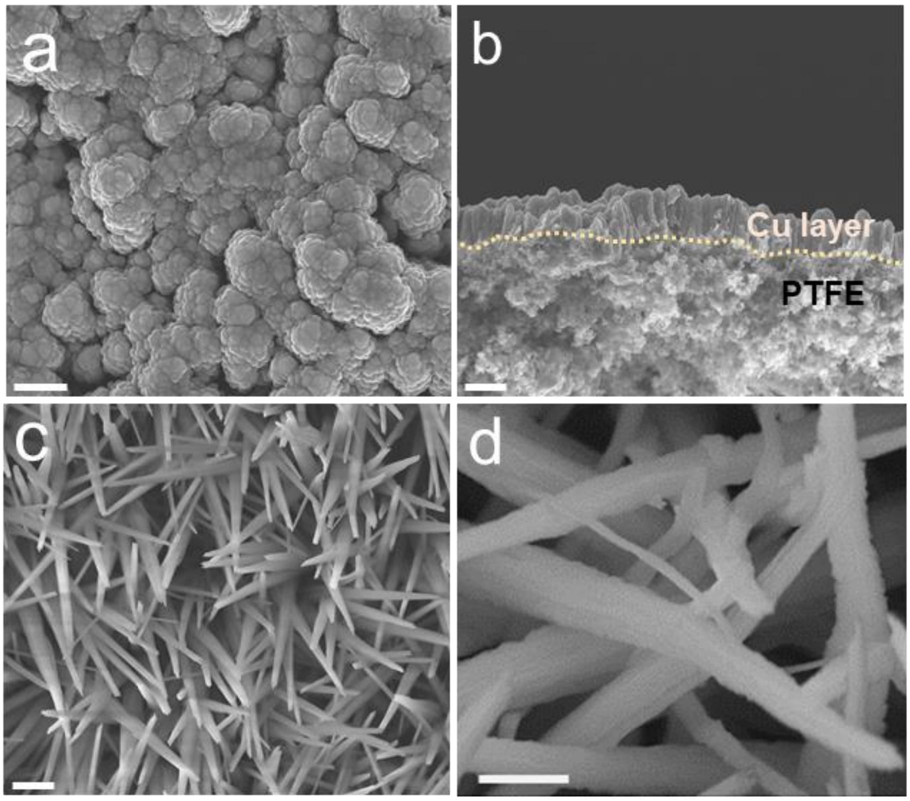

Supplementary Fig. 3. Microscopic characterizations of substrate. (**a**) Surface morphology and (**b**) cross-section view of sputtered Cu on the surface of GDE. Surface morphology of (**c**) anodized $Cu(OH)_2$ nanowires and (**d**) annealed CuO nanowires. Scale bars: 500 nm for (a), 1 μm for (b) and (c) and 400 nm for (d).

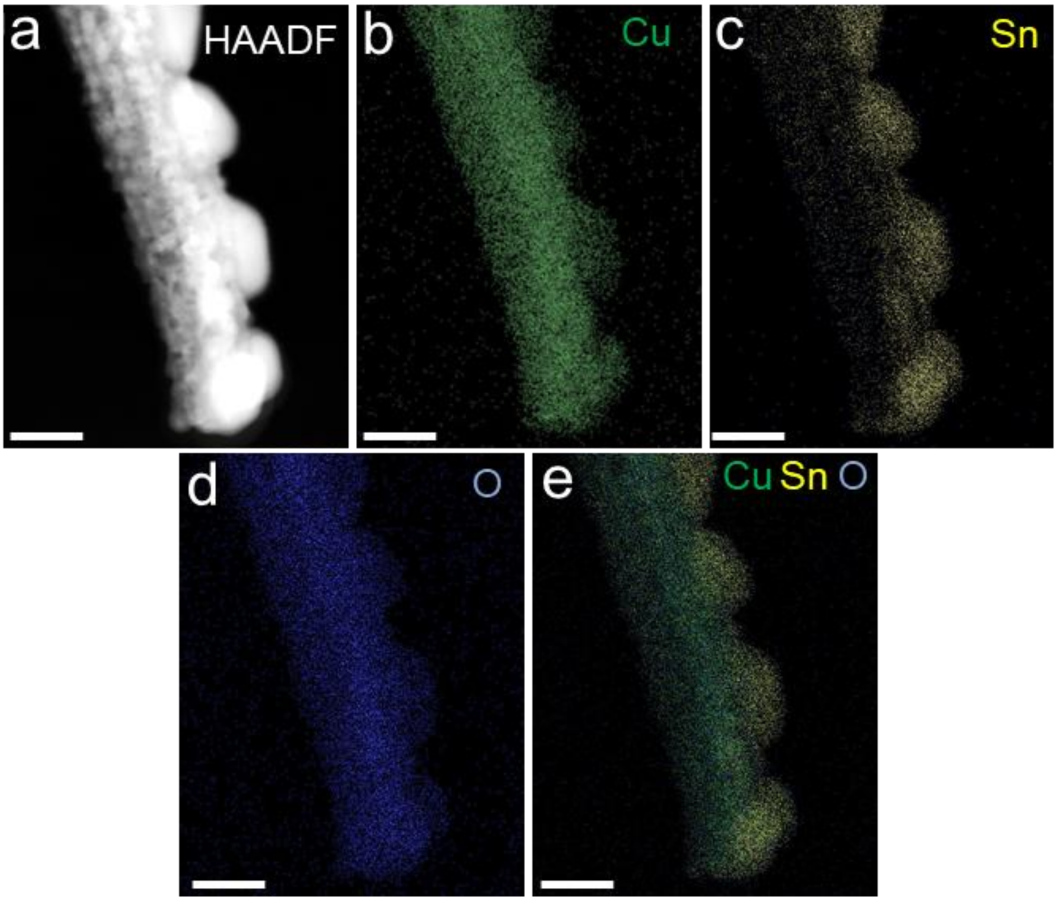

Supplementary Fig. 4. **Microscopic characterizations of as-prepared CuO-SnO2 SER sample.** (**a**) Representative HAADF (high-angle annular dark field) image of the as-prepared CuO-SnO2 SER with a sputtering thickness of 60 nm. Respective energy dispersive X-ray spectroscopic mapping of (**b**) Cu, (**c**) Sn, (**d**) O, (**e**) overlapped Cu, Sn and O. Scale bars: 50 nm for (a) to (e).

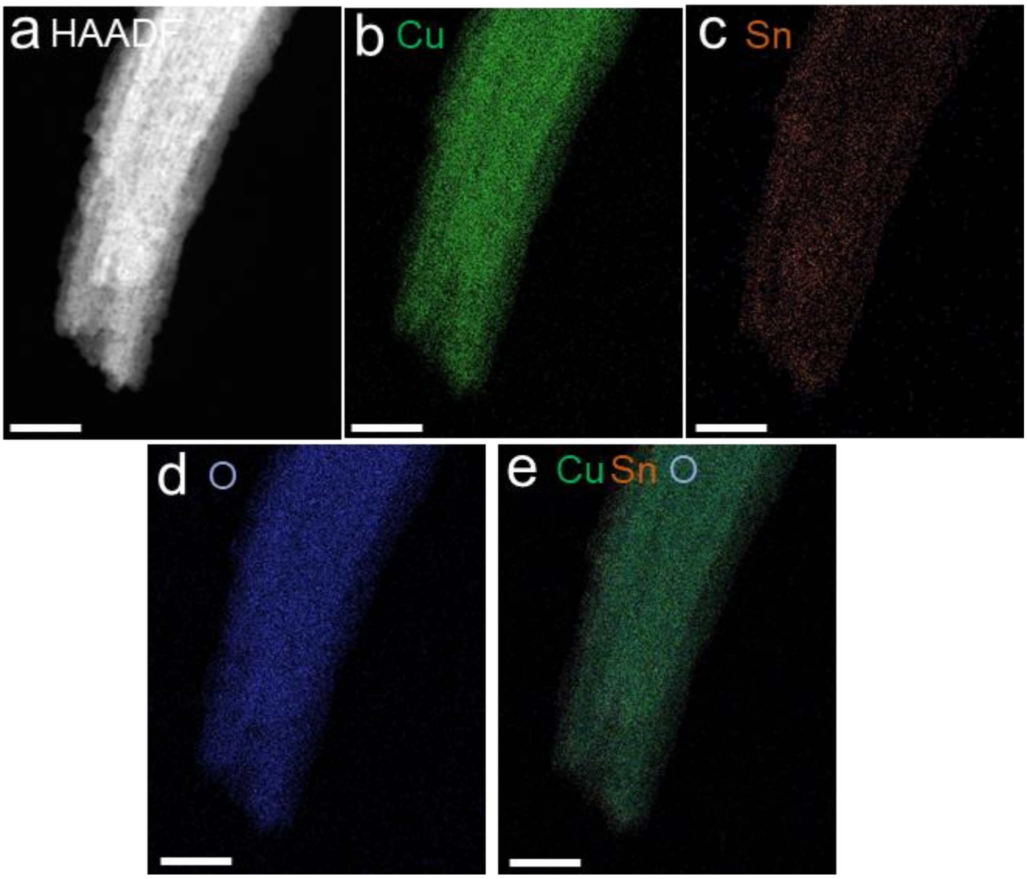

Supplementary Fig. 5. **Microscopic characterizations of as-prepared CuO-SnO_2 ALD sample.** (**a**) Representative HAADF (high-angle annular dark field) image of the as-prepared CuO-SnO_2 ALD with a thickness of ~1.35 nm for SnO_2 overlayer. Respective energy dispersive X-ray spectroscopic mapping of (**b**) Cu, (**c**) Sn, (**d**) O, (**e**) overlapped Cu, Sn and O. Scale bars: 50 nm for (a) to (e).

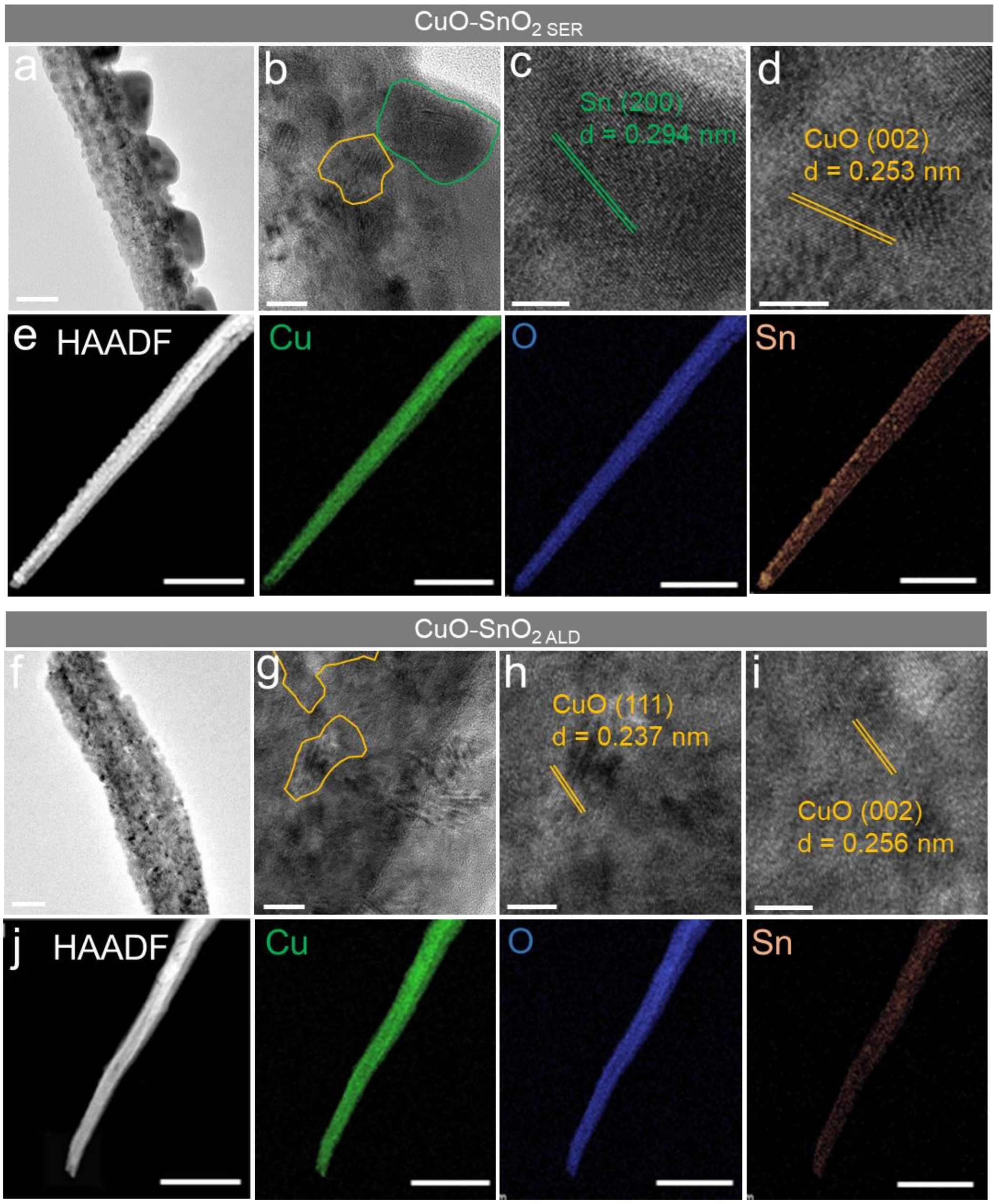

Supplementary Fig. 6. Microscopic characterizations of as-prepared CuO-SnO_2 samples. Representative (**a**) TEM and (**b**) HR-TEM images of the as-prepared CuO-$SnO_{2\ SER}$, lattice fringes in (**c**) and (**d**) are corresponded to Sn(200) and CuO(002) in the CuO-$SnO_{2\ SER}$ sample. (**e**) Representative HAADF image of the as-prepared CuO-$SnO_{2\ SER}$ and respective EDX spectroscopic mapping of Cu, O and Sn. Representative (**f**) TEM and (**g**) HRTEM images of the as-prepared CuO-$SnO_{2\ ALD}$, lattice fringes in (**h**) and (**i**) are corresponded to CuO(111) and CuO(002) in the CuO-$SnO_{2\ ALD}$ sample. (**j**) Representative HAADF image of the as-prepared CuO-$SnO_{2\ ALD}$ and respective EDX spectroscopic mapping of Cu, O and Sn. Scale bars: 50 nm for (a) and (f), 10 nm for (b) and (g), 5 nm for (c), (d), (h) and (i), 500 nm for (e) and (j).

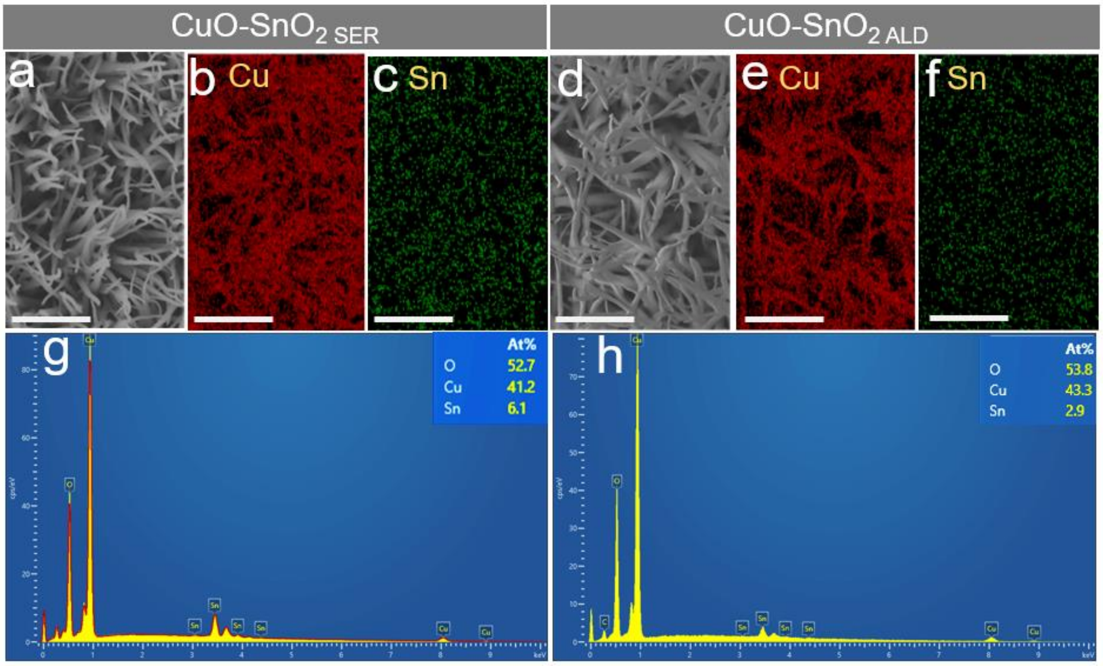

Supplementary Fig. 7. SEM-EDX analysis of as-prepared CuO-SnO_2 samples. (**a**) Representative image of the CuO-$SnO_{2\ SER}$ sample and the element distribution of (**b**) Cu and (**c**) Sn in the CuO-$SnO_{2\ SER}$ sample. (**d**) Representative image of the CuO-$SnO_{2\ ALD}$ sample and the element distribution of (**e**) Cu and (**f**) Sn in the CuO-$SnO_{2\ ALD}$ sample. Representative EDX spectrum for (**g**) CuO-$SnO_{2\ SER}$ and (**h**) CuO-$SnO_{2\ ALD}$. Scale bars: 3 μm for (a) to (f).

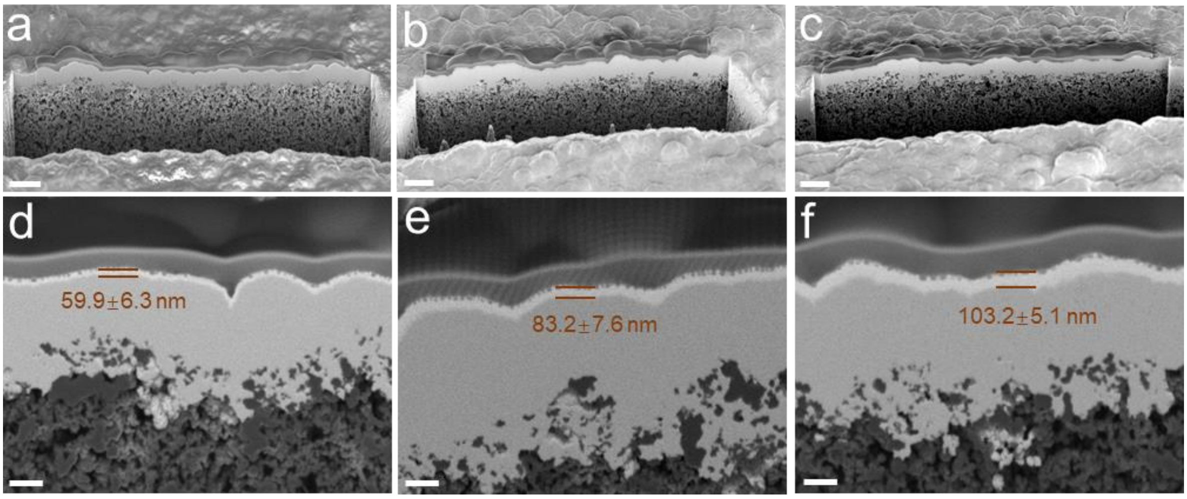

Supplementary Fig. 8. FIB cross-sectional view of the planar-CuO-supported ALD-SnO_2 layer with different deposition cycles. (**a**) and (**d**): 200 cycles; (**b**) and (**e**): 300 cycles; (**c**) and (**f**): 400 cycles. Scale bars: 2 μm for (a) to (c) and 400 nm for (d) to (f).

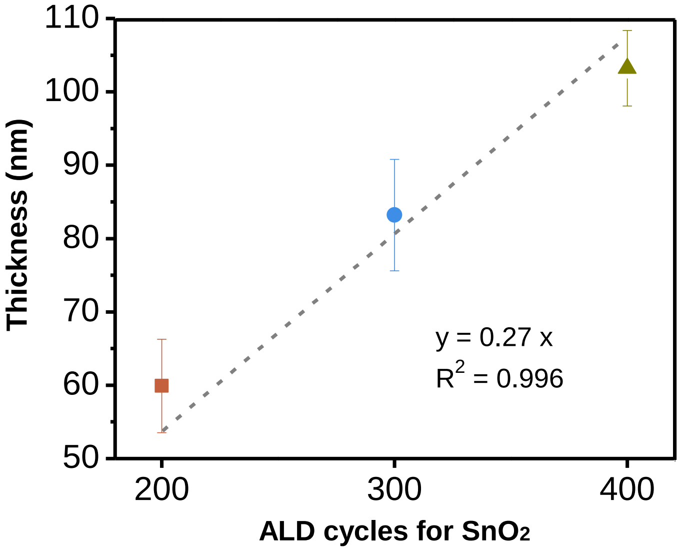

Supplementary Fig. 9. Calibration of thickness of ALD-SnO_2 layer. Calibration curve of the average thickness of ALD-SnO_2 layer as a function of the deposition cycles, the thickness is assessed from the FIB measurements shown in Supplementary Fig. 8. Since one cycle deposition corresponds to a thickness of 0.27 nm, the thickness of 5-ALD-cycle SnO_2 film was estimated to be 1.35 nm.

S2.2 Additional characterization data for electrocatalysts after reduction

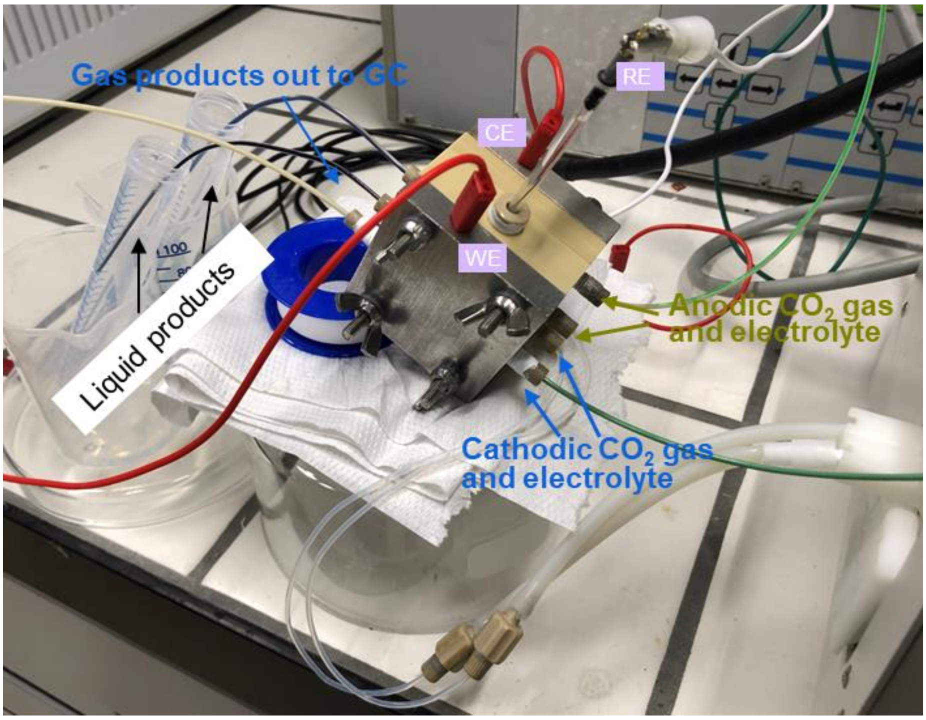

Supplementary Fig. 10. A photograph of the custom-built electrochemical flow cell. The flow cell shown here is used for all the electrochemical measurements in this study, including pre-reduction of catalysts, CO_2 electroreduction, double layer capacitance measurement, Pb underpotential deposition and CO adsorption on the catalyst.

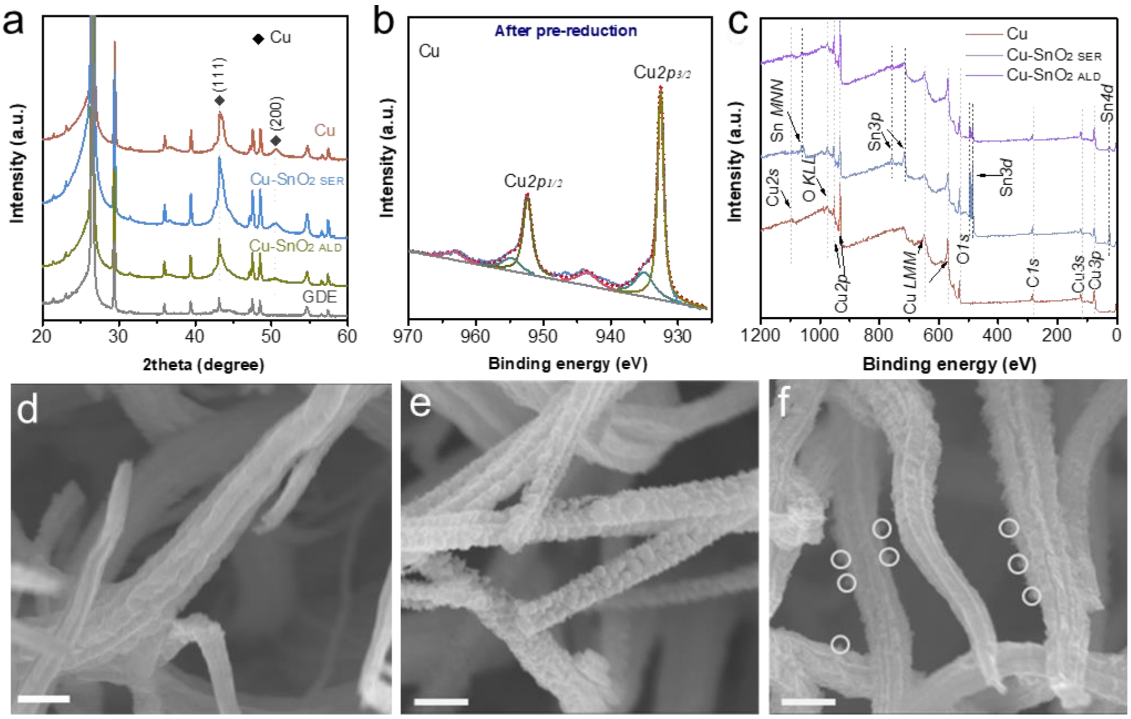

Supplementary Fig. 11. Structural and chemical characterizations of catalysts. (**a**) X-ray diffractograms of GDE substrate and Cu, Cu-SnO_2 SER and Cu-SnO_2 ALD catalysts after pre-reduction at -30 mA cm^{-2} for ~100 s. (**b**) High-resolution XPS spectrum of Cu after pre-reduction at -30 mA cm^{-2} for ~100 s. (**c**) XPS survey scan of Cu, Cu-SnO_2 SER and Cu-SnO_2 ALD catalysts after pre-reduction at -30 mA cm^{-2} for ~100 s. Representative SEM images of (**d**) Cu, (**e**) Cu-SnO_2 SER and (**f**) Cu-SnO_2 ALD catalysts after pre-reduction at -30 mA cm^{-2} for ~100 s. Scale bars: 200 nm for (c) to (f). The circular shape in (f) represents the appearance of SnO_2 based nanoparticles.

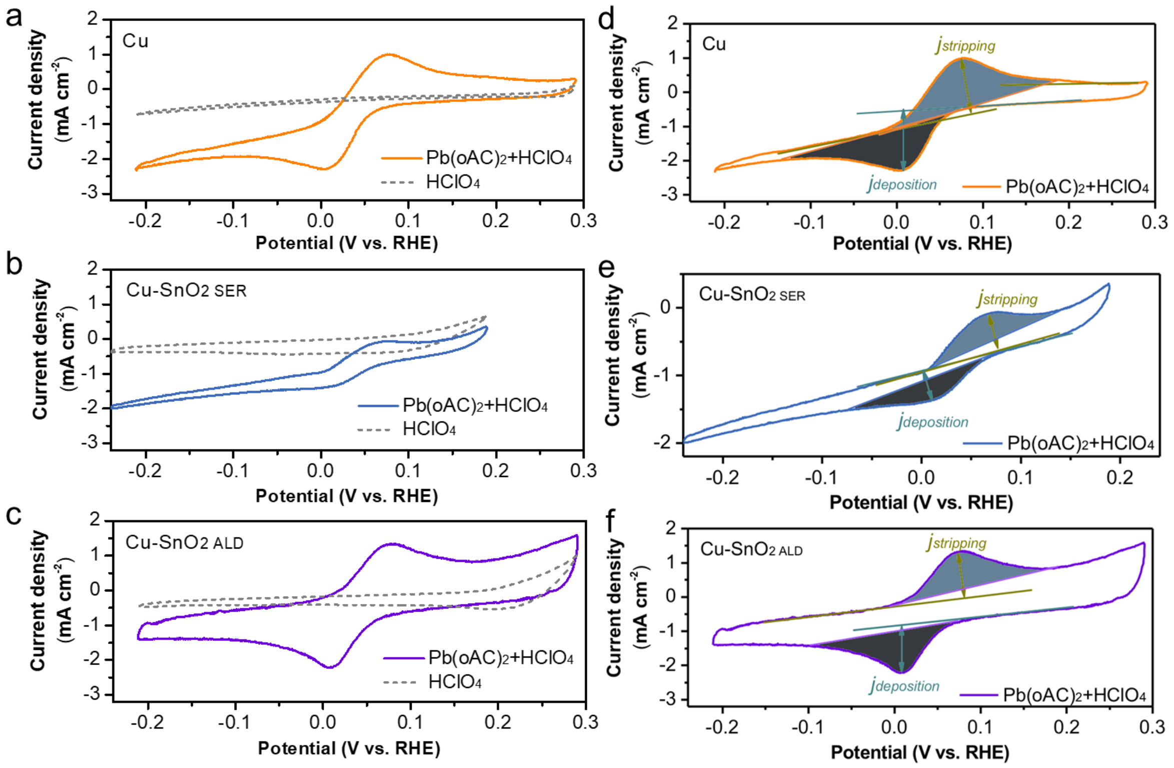

Supplementary Fig. 12. Pb underpotential deposition and stripping features on Cu active site. Representative cyclic voltammograms of Pb UPD and stripping on Cu active sites from (**a**) Cu, (**b**) Cu-SnO_2 SER and (**c**) Cu-SnO_2 ALD catalysts using the electrolyte containing 0.1 M $HClO_4$ and 0.001 M $Pb(OAc)_2$, the scan rate is 50 mV s^{-1} for all the voltammogram tests. (**d**)-(**f**) The corresponding cyclic voltammograms of three catalysts showing the details of underpotential deposition and stripping features on Cu active sites and the areas of the labeled shadows are the integrated charges summarized in Supplementary Table 2. The measurements were carried out in the same flow cell used for performing CO_2 reduction (Supplementary Fig. 10). During each measurement, different electrolytes and He gas were introduced into the corresponding chambers at a flow rate of 0.25 cm^3 min^{-1} and 20 cm^3 min^{-1}, respectively. The slanted baseline in these figures may be caused by the carbon component and the residual oxygen species inside the pores of GDE. In addition, we performed Pb UPD on the bare sputtered Sn electrode, showing no feature arose from UPD or stripping of Pb on Sn sites. This rules out the possible interference from Sn on the Pb monolayer formation on the Cu active sites.

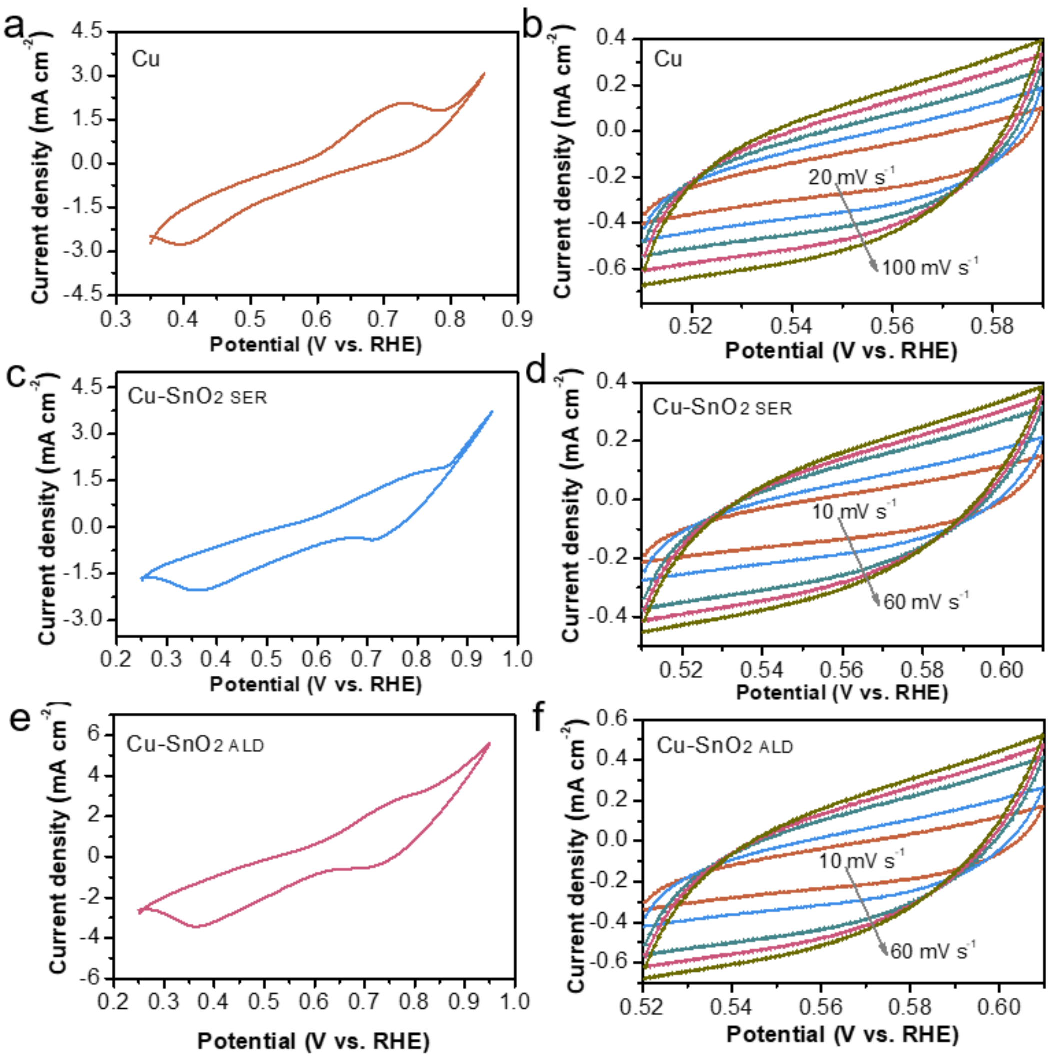

Supplementary Fig. 13. ECSA measurement of catalysts. Representative cyclic voltammograms on (**a**) Cu, (**c**) Cu-SnO_2 SER and (**e**) Cu-SnO_2 ALD catalysts measured from 0.35 to 0.85 V vs. RHE for Cu and 0.25 to 0.95 V vs. RHE for Cu-SnO_2 SER and Cu-SnO_2 ALD catalysts. Representative cyclic voltammograms on (**b**) Cu, (**d**) Cu-SnO_2 SER and (**f**) Cu-SnO_2 ALD catalysts within a non-faradaic current window. The curves were scanned at different sweep rates of 20, 40, 60, 80 to 100 mV s^{-1} for Cu, 10, 20, 40, 50, 60 mV s^{-1} for Cu-SnO_2 SER and Cu-SnO_2 ALD catalysts. The measurements were carried out in the same flow cell used for performing CO_2 reduction (Supplementary Fig. 10), with 0.5 M $KHCO_3$ and He being introduced into the cathodic liquid and gas chamber at flow rates of 0.25 and 20 cm^3 min^{-1}, respectively. The slanted baseline in these figures may be caused by the carbon component and the residual oxygen species inside the pores of GDE.

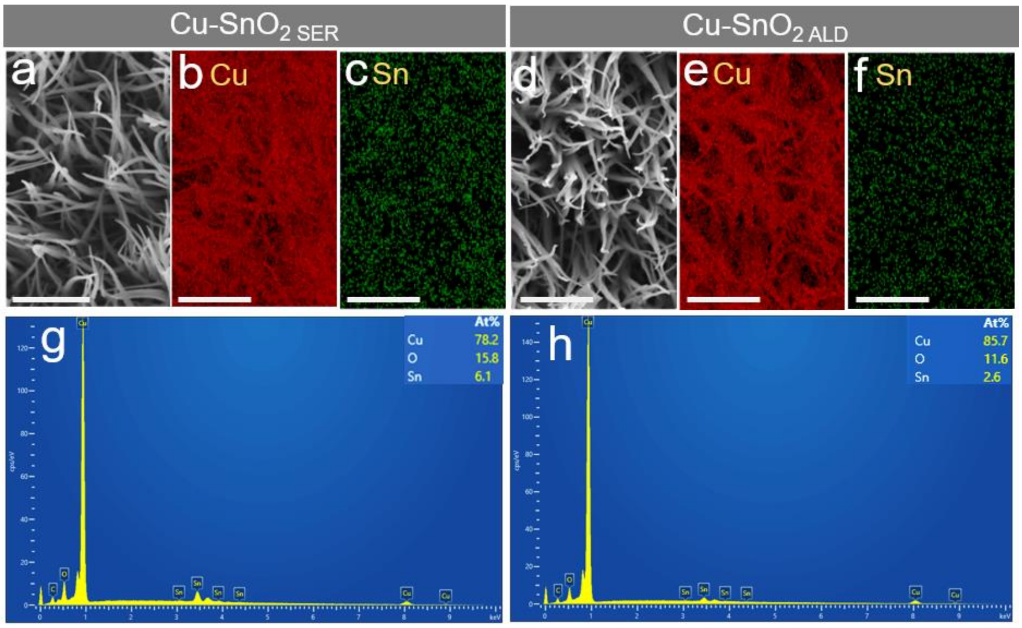

Supplementary Fig. 14. SEM-EDX analysis of pre-reduced Cu-SnO_2 catalysts. SEM-EDX mapping of Cu-SnO_2 SER and Cu-SnO_2 ALD after pre-reduction at a constant current density of -30 mA cm^{-2} for ~100 s in 0.5 M $KHCO_3$ electrolyte. (**a**) and (**d**): Microstructur. (**b**) and (**e**): Element mapping of Cu. (**c**) and (**f**) Element mapping of Sn. EDX spectrum detected on (**g**) Cu-SnO_2 SER and (**h**) Cu-SnO_2 ALD. Scale bars: 3 μm for (a) to (f).

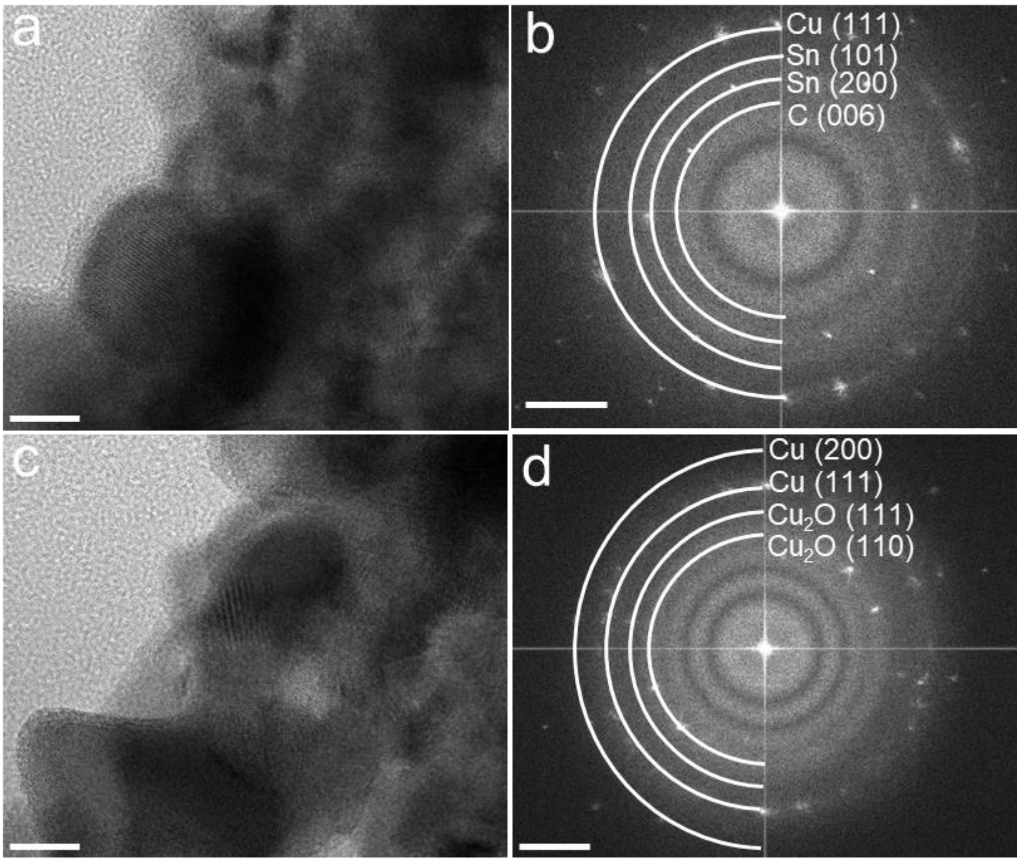

Supplementary Fig. 15. Structural characterizations of pre-reduced Cu-SnO$_2$ catalysts. (**a**) High-resolution TEM image of Cu-SnO$_{2\ SER}$ and (**b**) SAED pattern of Cu-SnO$_{2\ SER}$. (**c**) High-resolution TEM image of Cu-SnO$_{2\ ALD}$ and (**d**) SAED pattern of Cu-SnO$_{2\ ALD}$. Scale bars: 10 nm for (a) and (c), 2 nm^{-1} for (b) and (d).

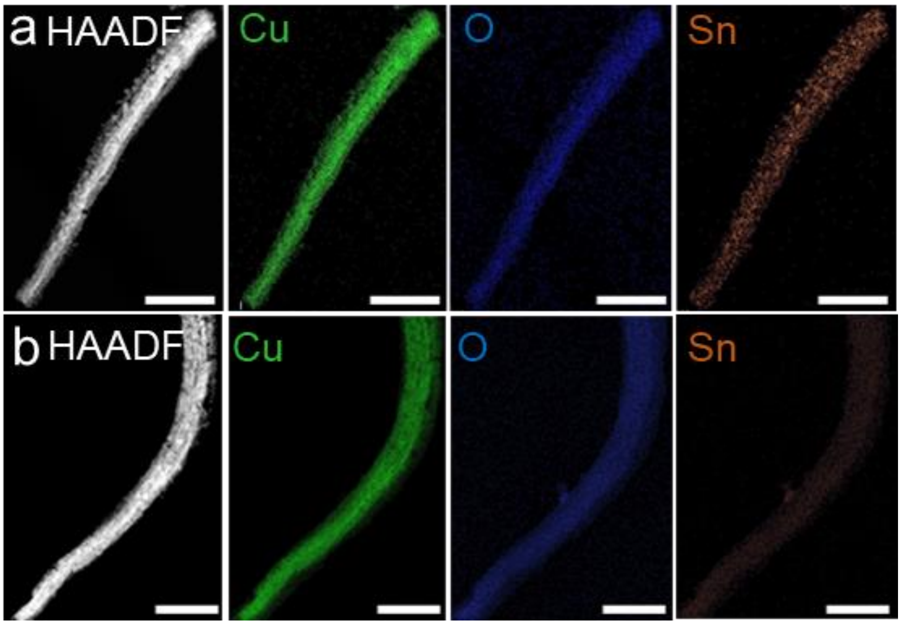

Supplementary Fig. 16. HAADF-EDX analysis of pre-reduced Cu-SnO_2 catalysts. The representative HAADF image and the EDX mapping of Cu, O and Sn of (**a**) Cu-$SnO_{2\ SER}$ and (**b**) Cu-$SnO_{2\ ALD}$ after pre-reduction. Scale bars: 500 nm (a), 200 nm for (b).

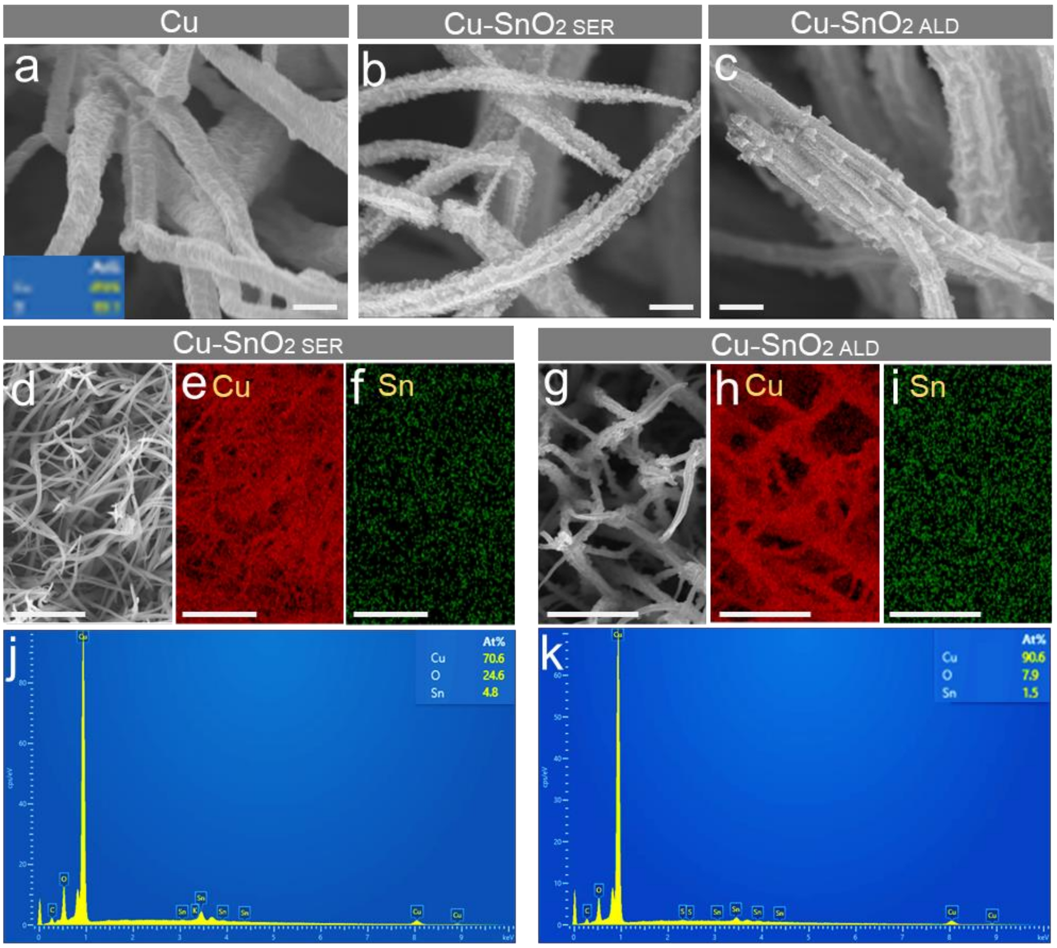

Supplementary Fig. 17. Structural characterization of Cu and Cu-SnO_2 catalysts after CO_2 electroreduction under -50 mA cm^{-2} for 50 min in 0.5 M $KHCO_3$ electrolyte. Scanning electron micrographs of (**a**) Cu, (**b**) Cu-SnO_2 SER and (**c**) Cu-SnO_2 ALD after 50 min-CO_2 reduction. SEM-EDX mapping of Cu and Sn elements in Cu-SnO_2 SER and Cu-SnO_2 ALD catalysts: (**d**) and (**g**) microstructure; (**e**) and (**h**) element mapping of Cu, (**f**) and (**i**) element mapping of Sn. and EDX spectrum detected on (**j**) Cu-SnO_2 SER and (**k**) Cu-SnO_2 ALD catalysts. Scale bars: 200 nm for (a) to (c), 3 μm for (d) to (f), 1.5 μm for (g) to (i). The nanowire features of Cu substrate remain unaltered and Cu-SnO_2 SER shows no observable morphological difference after 50-min CO_2 reduction. Interestingly, Cu-SnO_2 ALD undergoes reconstruction, forming SnO_2 nanoparticles/nanocubes with a size of 50 to 100 nm on the surface of Cu nanowires.

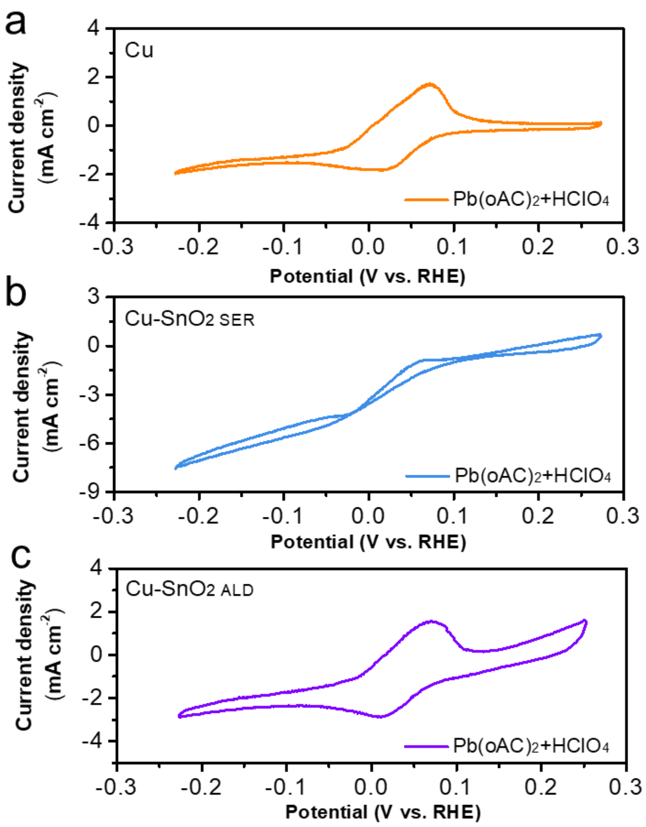

Supplementary Fig. 18. Pb UPD and stripping features on Cu active site after 50 min-CO_2 reduction at -50 mA cm^{-2}. Representative cyclic voltammograms showing the UPD and stripping of Pb on Cu active sites from (**a**) Cu nanowires, (**b**) Cu-SnO_2 SER and (**c**) Cu-SnO_2 ALD catalysts. The scan rate is 50 mV s^{-1} for all the voltammogram tests. The measurements were carried out in the same flow cell used for performing CO_2 reduction, with the electrolytes and He gas being admitted into the corresponding chambers at a flow rate of 0.25 cm^3 min^{-1} and 20 cm^3 min^{-1}, respectively.

S2.3 Additional electrochemistry data for Cu and Cu-Sn catalysts in 0.5 M $KHCO_3$

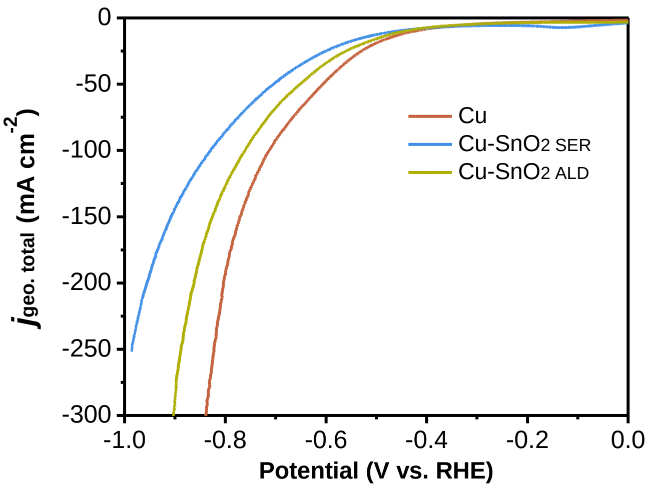

Supplementary Fig. 19. Electrochemical measurement of catalysts. Representative linear sweep voltammograms of Cu, Cu-SnO_2 SER and Cu-SnO_2 ALD catalysts in 0.5 M $KHCO_3$ electrolyte, with a scan rate of 25 mV s^{-1}.

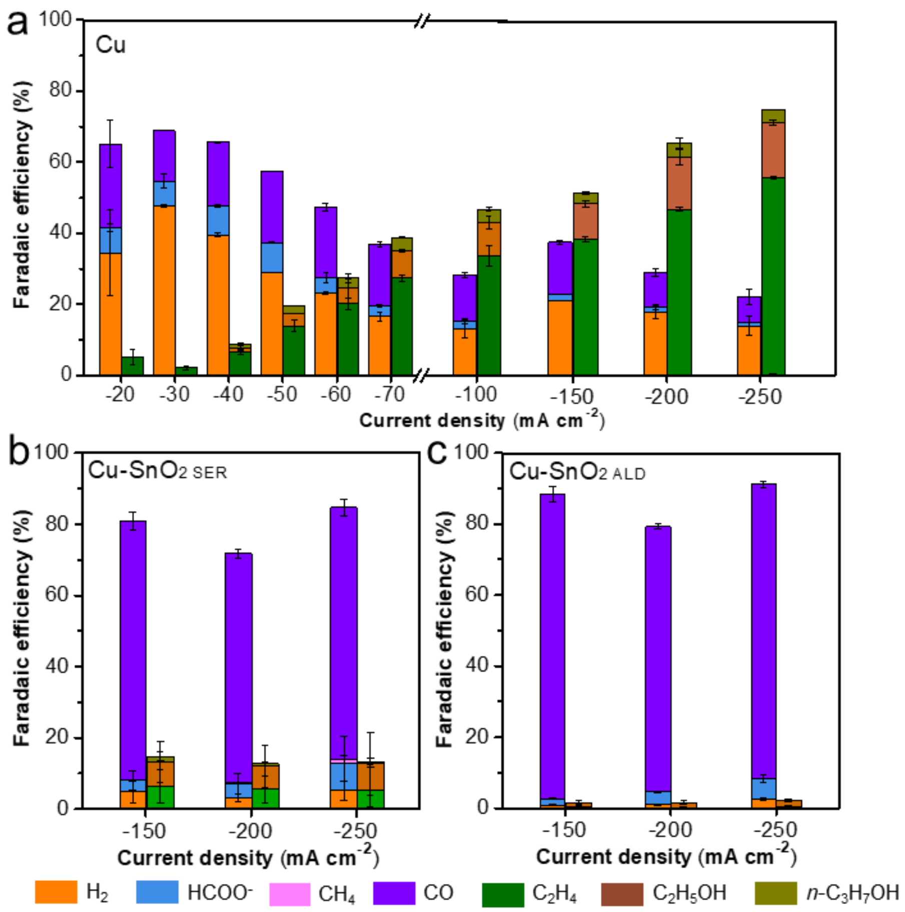

Supplementary Fig. 20. Selectivity towards CO_2 reduction products on catalysts at different current densities. (**a**) Faradic efficiency of major products on Cu cathodes at different current densities. Faradic efficiency of major products on (**b**) Cu-SnO_2 SER and (**c**) Cu-SnO_2 ALD catalysts under higher current densities of -150, -200 and -250 mA cm^{-2}. Each data point in (a) to (c) corresponds to the average value of three independent measurements and the error bars represent the standard deviations of these measurements.

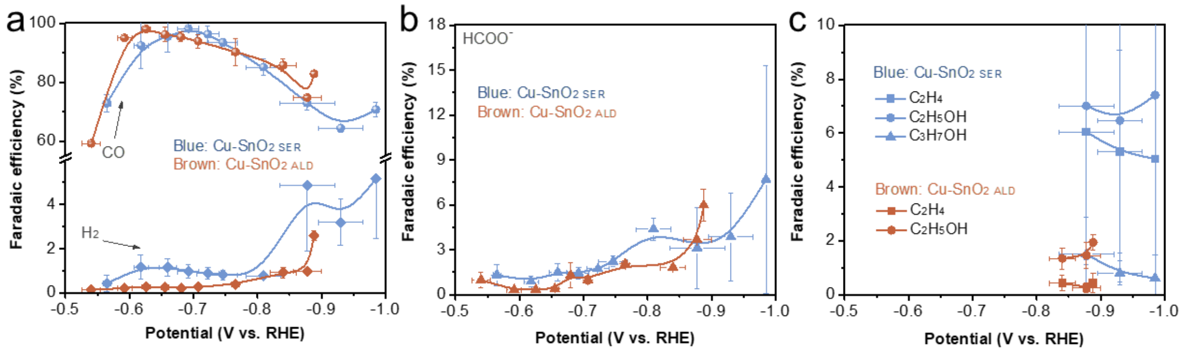

Supplementary Fig. 21. Selectivity towards CO_2 reduction products on catalysts at different potentials. Faradaic efficiency of (**a**) CO and H_2, (**b**) $HCOO^-$ and (c) C_2H_4, C_2H_5OH and C_3H_7OH generated on Cu-SnO_2 SER and Cu-SnO_2 ALD catalysts at different potentials.

S2.4 Electrocatalytic performance of Cu-Sn catalysts in 1 M KOH

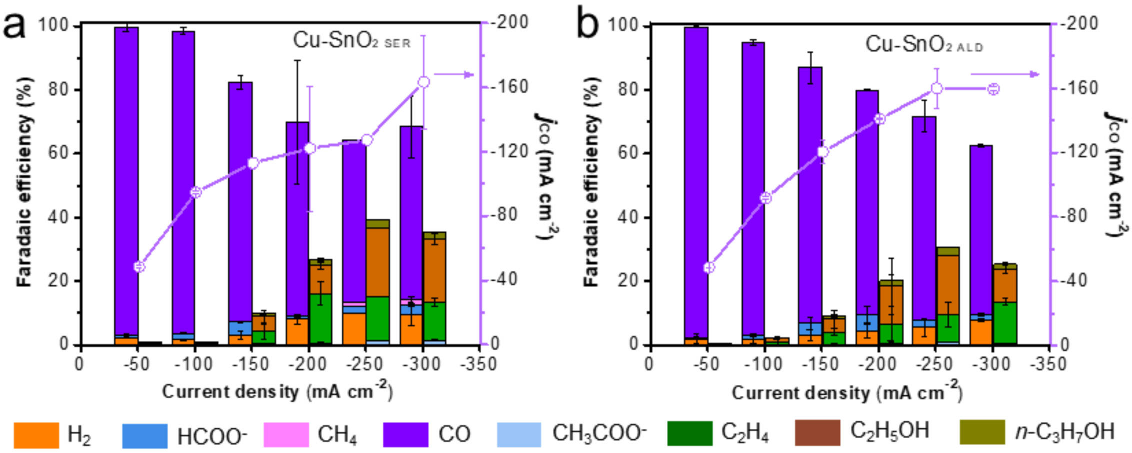

Supplementary Fig. 22. Selectivity towards CO_2 reduction products on Cu-SnO_2 catalysts in an alkaline electrolyte. Faradaic efficiency of products generated on (**a**) Cu-$SnO_{2\ SER}$ and (**b**) Cu-$SnO_{2\ ALD}$ catalysts under different current densities in 1 M KOH electrolyte. Each data point in (a) and (b) corresponds to the average value from three to four independent measurements and the error bars represent the standard deviations of these measurements. If the cathodic current density is lower than 100 mA cm^{-2}, both Cu-SnO_2 catalysts show the similar performance with the one observed in 0.5 M $KHCO_3$, with optimum FE_{CO} of around 97% being achieved at -50 mA cm^{-2}. Both catalysts deliver large quantities of C_{2+} products if the cathodic current density is larger than 150 mA cm^{-2}. The FE_{CO} drops to ~53% when the current density reaches -300 mA cm^{-2}. Note that electrolyte of KOH has a poorer buffer capability as compared to $KHCO_3$ solution. Thus an increase of the local pH accompanies the augmentation of the applied cathodic current density in 1 M KOH electrolyte[2]. This effect may favor the formation of C_2 products in 1 M KOH, as the C_2 products generated through *CO-*CO coupling is favored at higher local pH[3,4].

S2.5 Morphological and electrochemical characterization of planar CuO supported SnO_2 catalyst

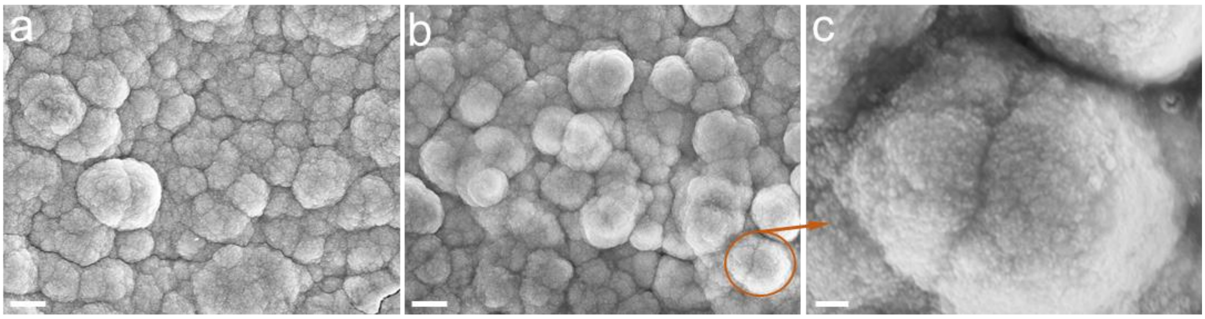

Supplementary Fig. 23. Morphological characterization of planar CuO substrate and ALD-SnO_2 coated substrate. Representative SEM images of (**a**) electrodeposited planar CuO film, (**b-c**) 5-ALD-cycle SnO_2 coated planar CuO. Scale bars: 1 μm for (a) and (b), 200 nm for (c).

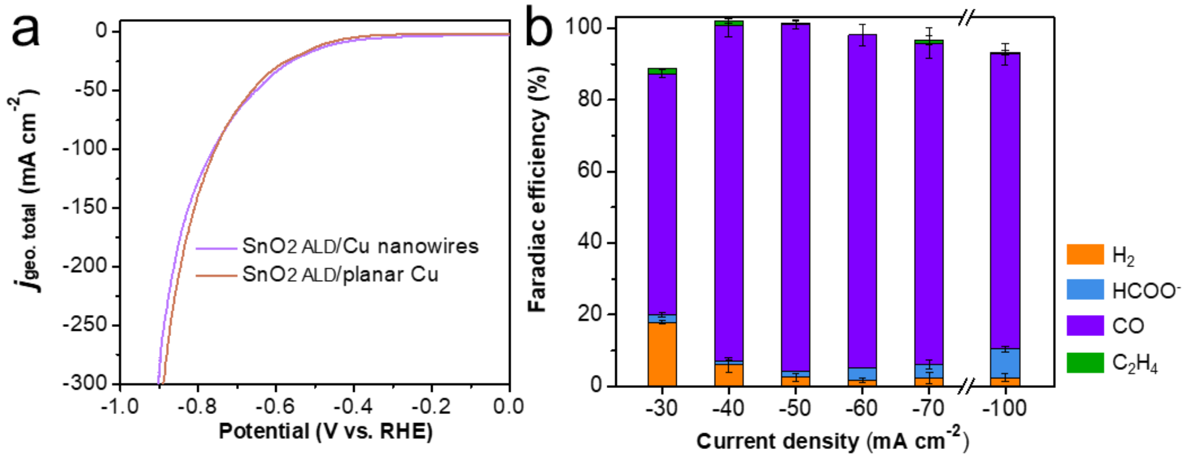

Supplementary Fig. 24. Catalytic performance of planar CuO supported SnO_2 catalyst. (**a**) Representative linear sweep voltammograms of CuO nanowires and planar CuO (CuO_p) supported SnO_2 catalysts (CuO_p-$SnO_{2\ ALD}$). (**b**) Faradic efficiencies of the products detected on CuO_p-$SnO_{2\ ALD}$ under different current densities in 0.5 M $KHCO_3$ electrolyte. Each data point in (b) corresponds to the average value from three to four independent measurements and the error bars represent the standard deviations of these measurements. Cu_p-$SnO_{2\ ALD}$ achieves a peak FE of ca. 97% towards CO generation at -50 mA cm^{-2} in 0.5 M $KHCO_3$ and FE_{CO} decreases to 82% if the cathodic current density is increased to 100 mA cm^{-2}. Additionally, the FE of $HCOO^-$ increased from 2% to 8% within the applied current densities, which is ×3~×5 higher than the one detected on Cu NWs supported $SnO_{2\ ALD}$ (Supplementary Table 11). Note that Cu_p-$SnO_{2\ ALD}$ catalyst shows almost the same linear sweep voltammetric feature with that of Cu-$SnO_{2\ ALD}$.

S2.6 Current against geometric surface area and electrochemical active surface area

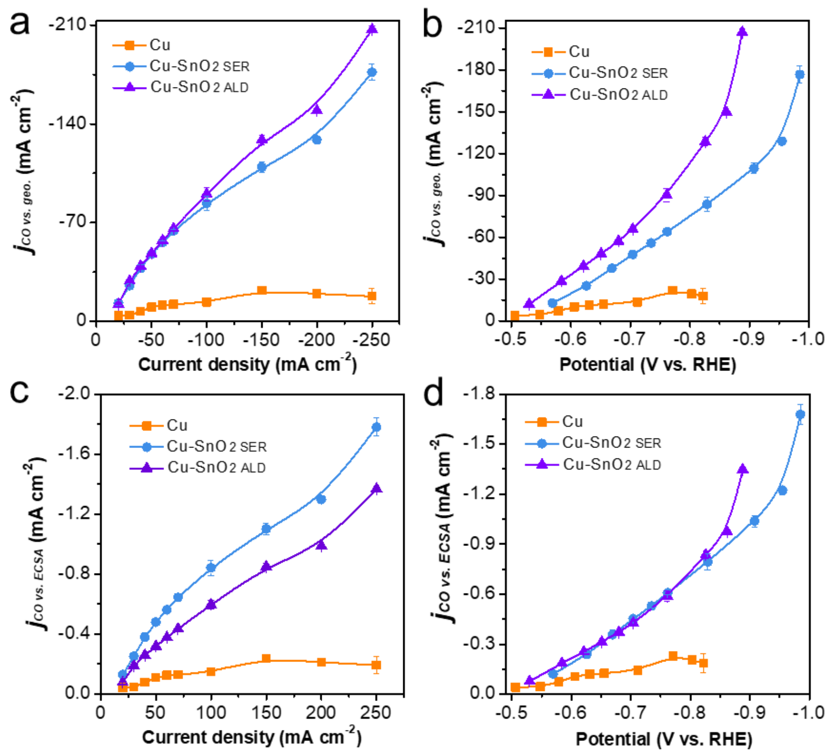

Supplementary Fig. 25. Activity of CO_2 reduction on three catalysts. The partial current density of CO normalized against (**a-b**) geometric surface area and (**c-d**) electrochemical surface area (ECSA) of three catalysts as a function of current density or applied potential. Each data point in (a)-(d) corresponds to the average value from three to four independent measurements and the error bars represent the standard deviations of these measurements.

S2.7 Effect of thickness of sputtered Sn and ALD-SnO_2 on the catalytic performance and morphology

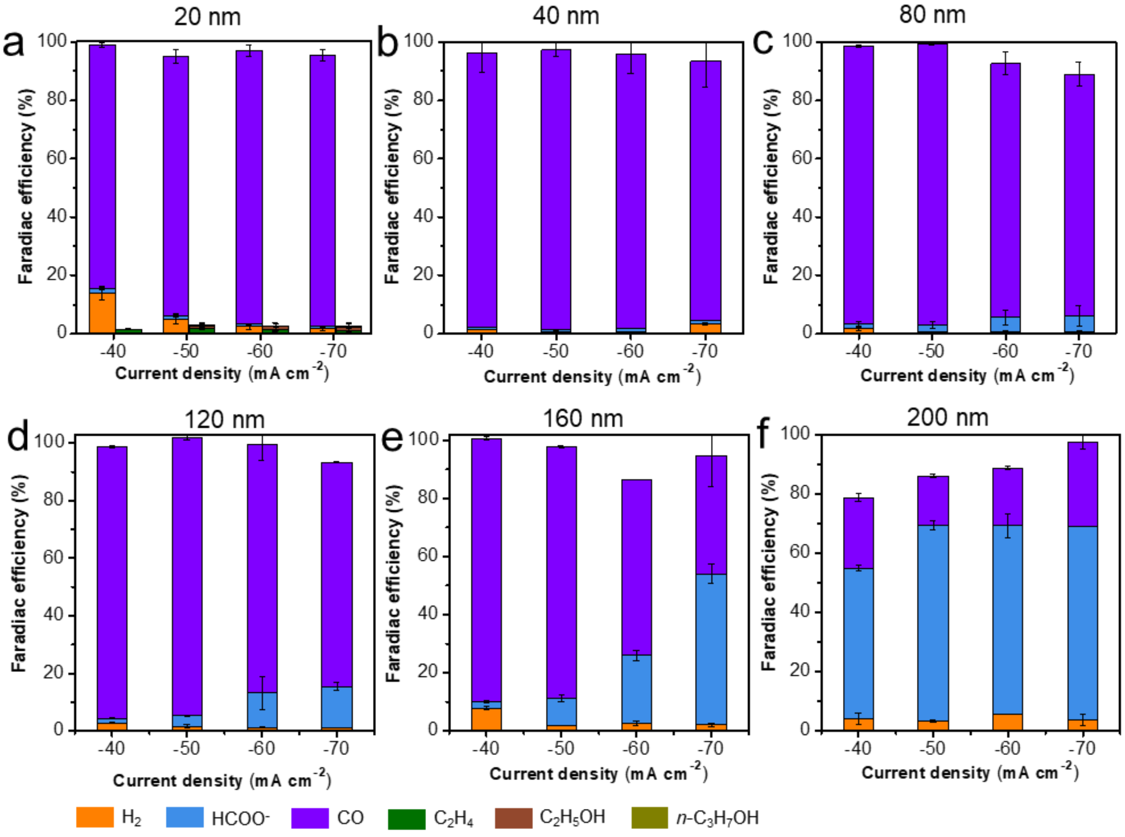

Supplementary Fig. 26. Selectivity of Cu-SnO_2 SER catalysts towards CO_2 reduction products. Faradic efficiency of major products on Cu-SnO_2 SER catalysts with different sputtered thickness of Sn: (**a**) 20 nm, (**b**) 40 nm, (**c**) 80 nm, (**d**) 120 nm, (**e**) 160 nm and (**f**) 200 nm. Each data point in (a)-(f) corresponds to the average value from two to three independent measurements and the error bars represent the standard deviations of these measurements.

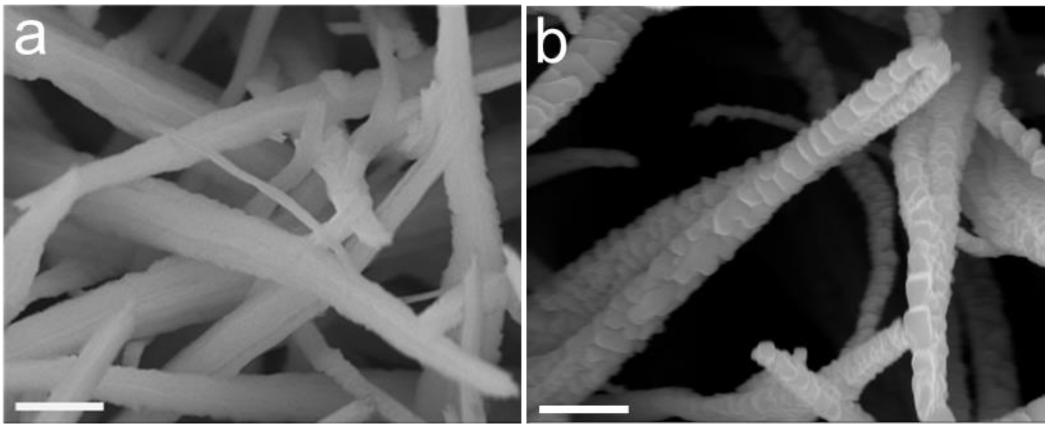

Supplementary Fig. 27. **Morphological characterization of Cu-SnO_2 SER catalyst.** Representative SEM images of as-prepared (**a**) CuO substrate and (**b**) CuO coated with 120 nm-thick sputtered Sn. Scale bars: 400 nm for (a) and (b).

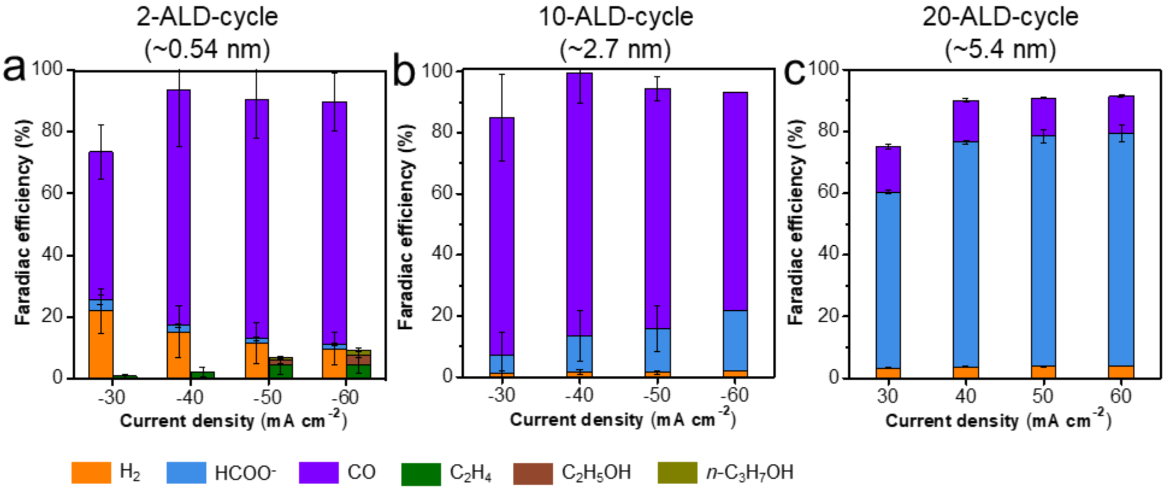

Supplementary Fig. 28. Selectivity of Cu-SnO2 ALD catalysts towards CO2 reduction products. Faradic efficiency of major products on Cu-SnO2 ALD catalysts with different cycles of atomic layer deposition of SnO2: (**a**) 2 cycles, (**b**) 10 cycles and (**c**) 20 cycles. Each data point in (a)-(c) corresponds to the average value from two to three independent measurements and the error bars represent the standard deviations of these measurements.

S2.8 Electrocatalytic performance of bare sputtered Sn and ALD-SnO_2 catalysts

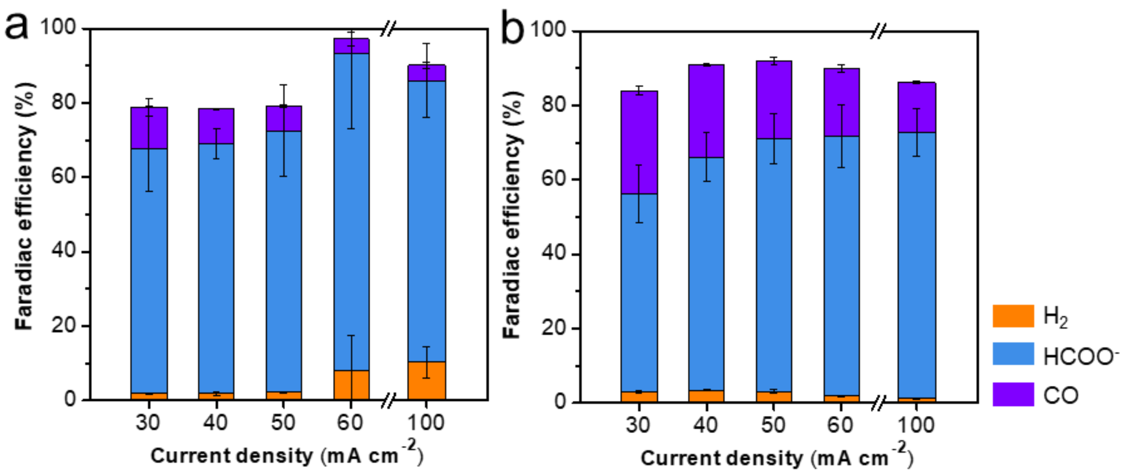

Supplementary Fig. 29. Selectivity of bare Sn and SnO_2 catalysts towards CO_2 reduction products. Faradic efficiency of major products detected on (**a**) pure 60 nm-thick Sn and (**b**) 300-ALD-cycle SnO_2 (corresponding to a thickness of ~81 nm) catalysts. Each data point in (a) and (b) corresponds to the average value from two to three independent measurements and the error bars represent the standard deviations of these measurements.

S2.9 Additional data for stability test

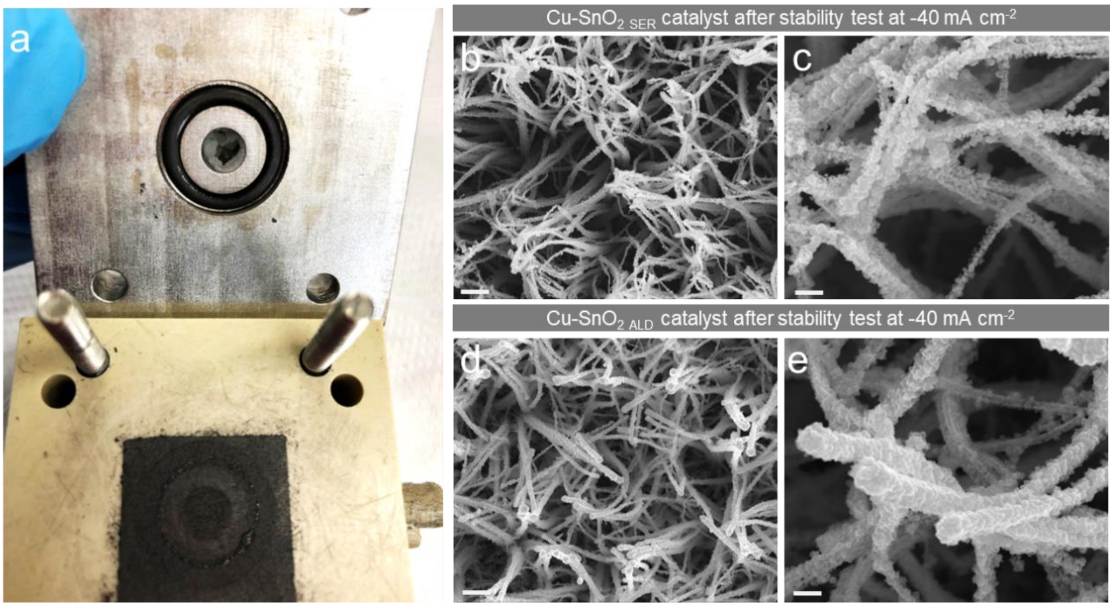

Supplementary Fig. 30. Morphological characterization of Cu-SnO_2 catalyst after stability measurement. (**a**) Representative photographs of the cathodic gas-flow plate and the back of GDE after 4.5-h CO_2 reduction at a current density of -40 mA cm^{-2}. Representative SEM images of (**b-c**) Cu-$SnO_{2\ SER}$ and (**d-e**) Cu-$SnO_{2\ ALD}$ after ~120 h-electrolysis at a current density of -40 mA cm^{-2}. Scale bars: 1 μm for (b) and (d), 200 nm for (c) and (e). Obvious salt precipitation appeared inside the cathodic gas chamber and small electrolyte drops were observed on the surface of carbon fiber.

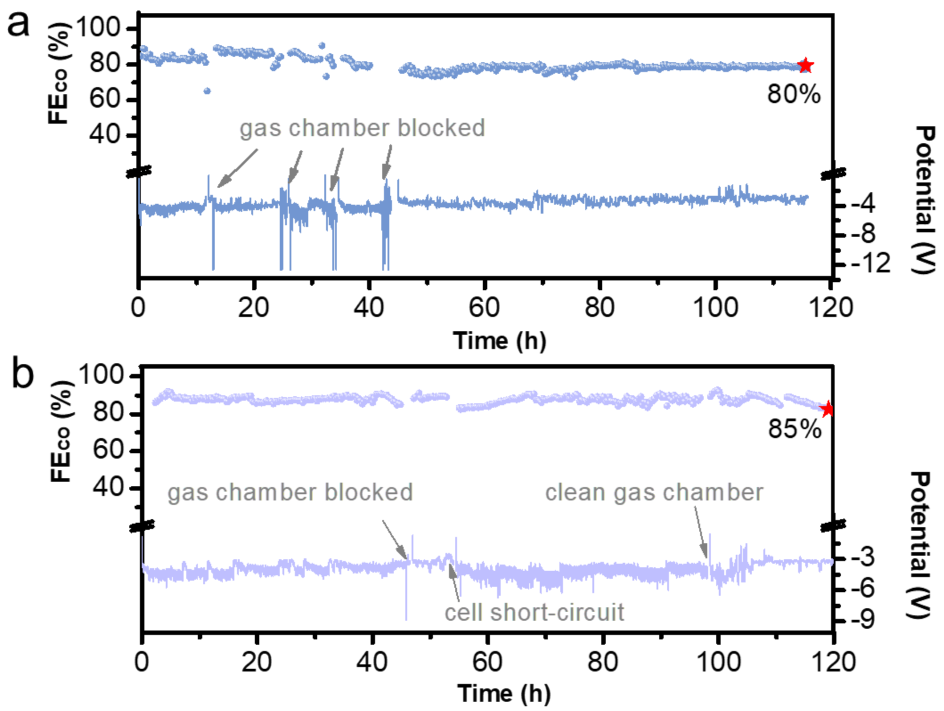

Supplementary Fig. 31. Stability test of Cu-SnO_2 catalyst at -100 mA cm^{-2}. Faradaic efficiency of produced CO and the detected half-cell potential at a current density of -100 mA cm^{-2} during 120-hr electrolysis on (**a**) Cu-SnO_2 SER and (**b**) Cu-SnO_2 ALD catalysts.

S2.10 Additional data for X-ray absorption spectroscopy

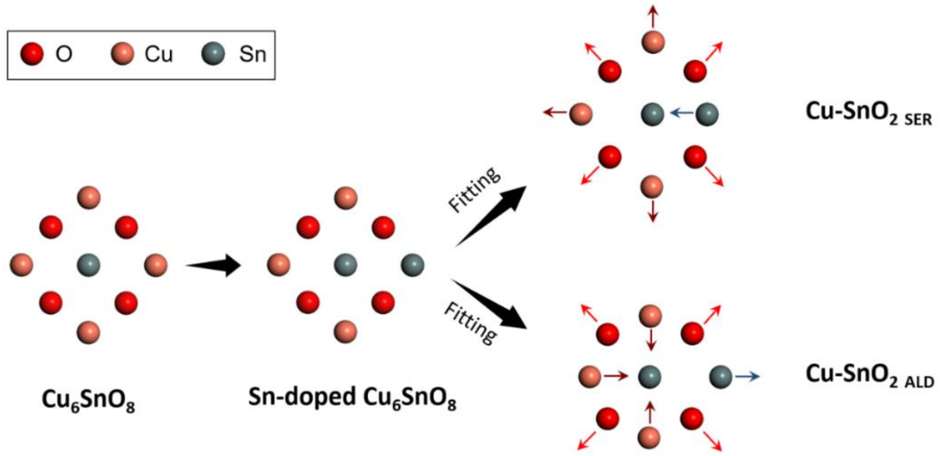

Supplementary Fig. 32. Schematics of model structure relaxations during EXAFS fittings. We applied the same structure (Sn-doped Cu_6SnO_8: https://materialsproject.org/materials/mp-1147658/) for the EXAFS fittings of both $Cu-SnO_2$ ALD and $Cu-SnO_2$ SER catalysts. Using the FEFF6 program (included in Demeter (v.0.9.26): https://bruceravel.github.io/demeter/), we found that the model structure underwent different relaxations to form Cu-Sn bond in the $Cu-SnO_2$ ALD catalyst and Sn-Sn bond in the $Cu-SnO_2$ SER catalyst, respectively.

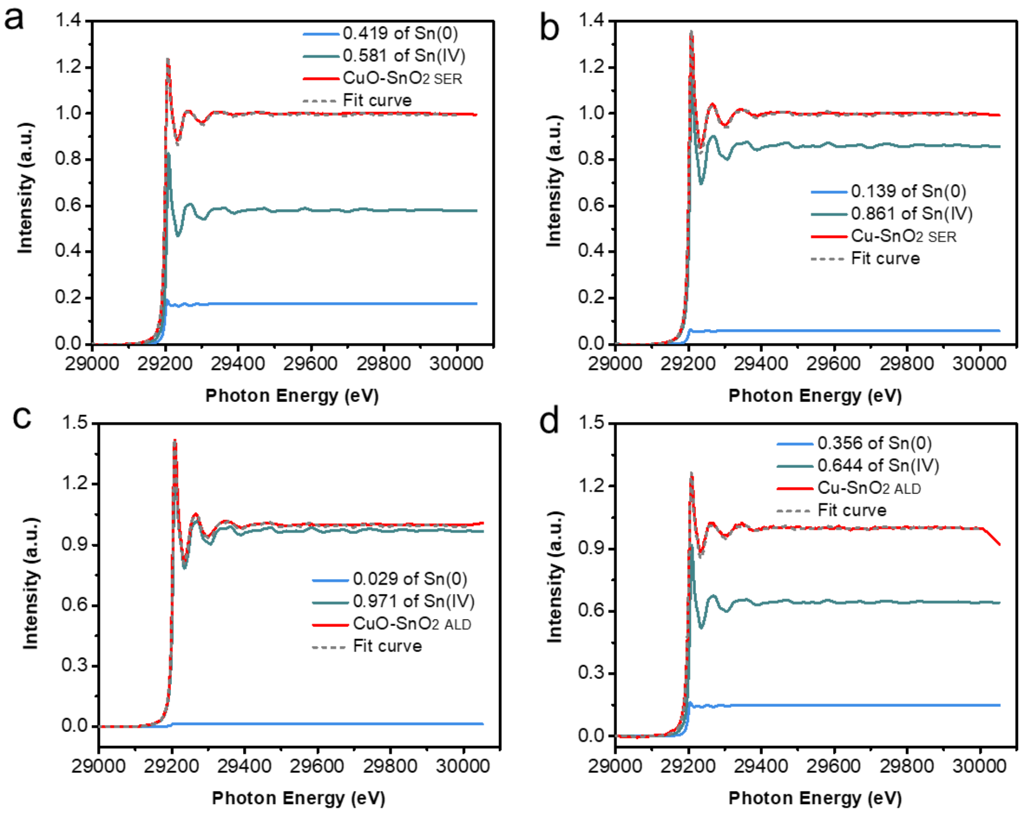

Supplementary Fig. 33. X-ray absorption spectroscopy analysis of $Cu-SnO_2$ catalysts. Linear combination fitting analysis at the Sn K-edge XAS of (**a**) $CuO-SnO_2$ SER, (**b**) $Cu-SnO_2$ SER, (**c**) $CuO-SnO_2$ ALD and (**d**) $Cu-SnO_2$ ALD catalysts.

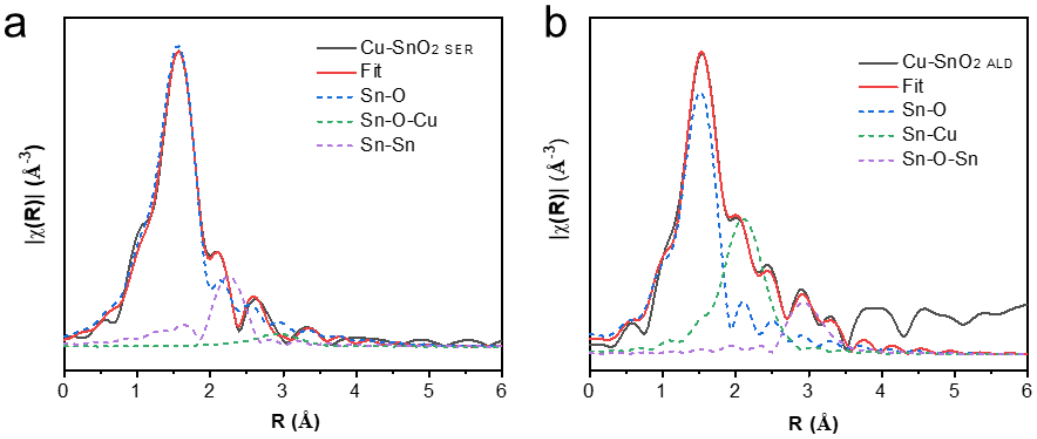

Supplementary Fig. 34. X-ray absorption spectroscopy analysis of Cu-SnO_2 catalysts. The Sn K-post-edge XAS fitting results for (**a**) Cu-SnO_2 SER and (**b**) Cu-SnO_2 ALD catalysts at a R range between 0.5 and 3.3 Å.

S2.11 Additional data for CO adsorption

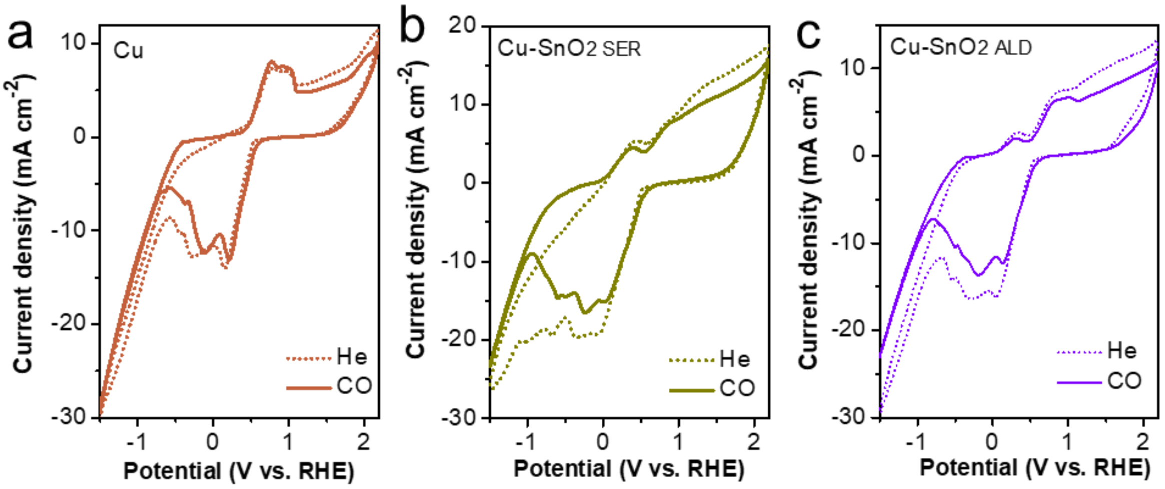

Supplementary Fig. 35. Cyclic voltammetry of three catalysts. Cyclic voltammograms measured on (**a**) Cu substrate, (**b**) Cu-SnO_2 SER and (**c**) Cu-SnO_2 ALD catalysts in the flow cell, with 0.5 M $KHCO_3$ and CO or He being admitted at flow rates of 0.25 and 50 cm^3 min^{-1}, respectively. The scan rate is 50 mV s^{-1} for all the CV measurements.

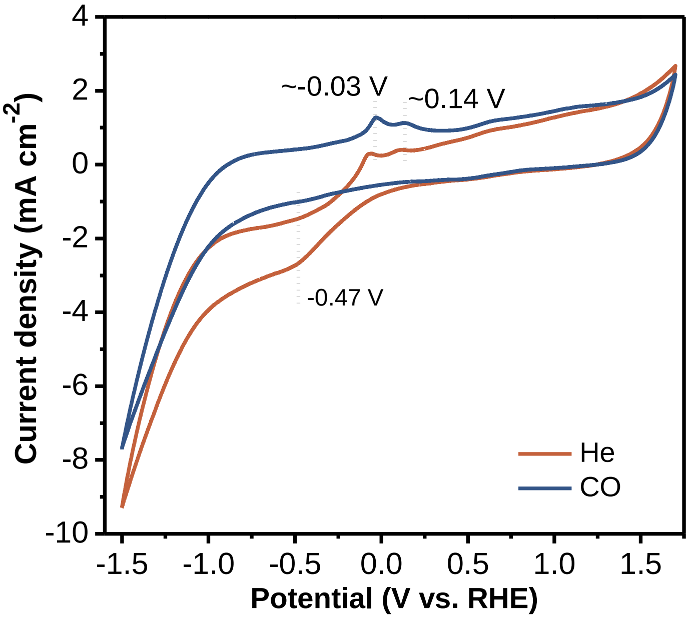

Supplementary Fig. 36. Cyclic voltammetry of bare Sn catalysts. Cyclic voltammograms measured on bare sputtered Sn electrode in the flow cell, with 0.5 M $KHCO_3$ and CO or He being introduced at flow rates of 0.25 and 50 cm^3 min^{-1}, respectively. The scan rate is 50 mV s^{-1}.

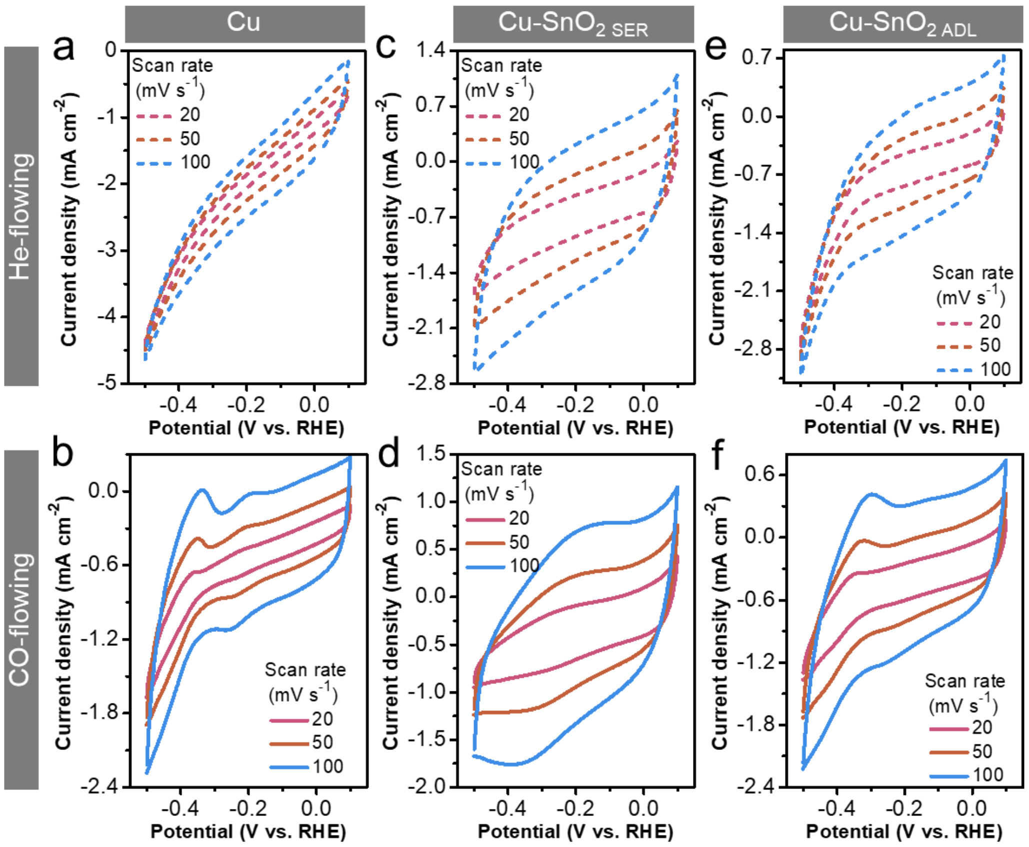

Supplementary Fig. 37. CO adsorption on Cu and Cu-SnO2 catalysts. Cyclic voltammograms measured on (**a, b**) Cu substrate, (**c, d**) Cu-SnO_2 SER and **(e, f**) Cu-SnO_2 ALD catalysts under He (dash line) and CO flowing (solid line) condition.

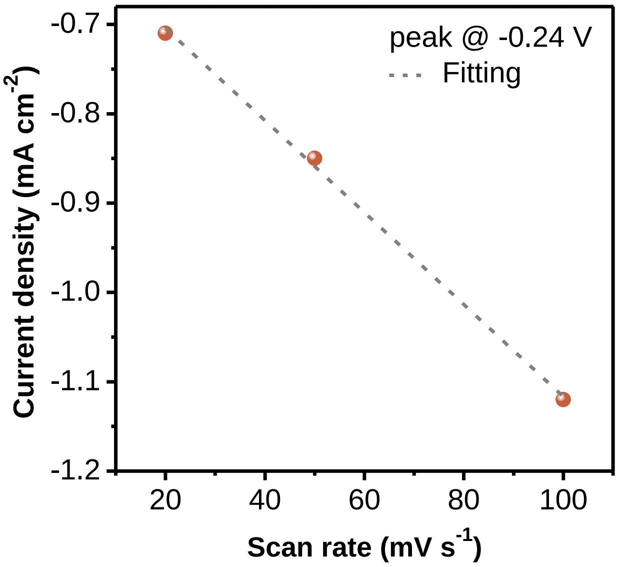

Supplementary Fig. 38. The current density of the voltametric peaks at -0.24 V vs. RHE measured on Cu surface against the scant rate. All the data is extracted from Supplementary Fig. 37b.

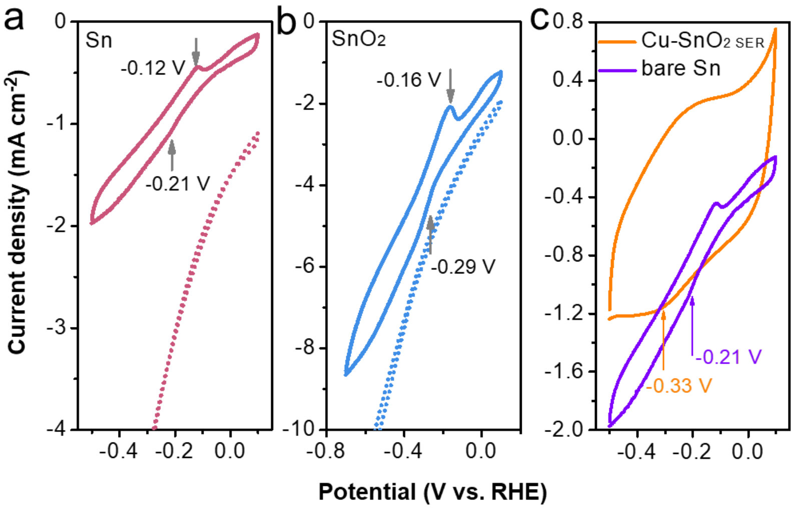

Supplementary Fig. 39. CO adsorption on bare Sn and Cu-SnO_2 SER catalysts. (**a, b**) Cyclic voltammograms measured on pure sputtered Sn (~60 nm) and ALD-SnO_2 (~81 nm) catalysts within a small potential window from -0.5 to 0.1 V vs. RHE for Sn and -0.7 to 0.1 V for SnO_2. He (dash line) or CO (solid line) gas was continuously flowed into the reactor at 50 cm^3 min^{-1} during each measurement. (**c**) Comparison of the cyclic voltammograms of Cu-SnO_2 SER catalyst and bare Sn electrode. The scan rate for all the CV curves is 50 mV s^{-1}. The integrated charge for CO desorption peak on Cu-SnO_2 SER is 0.18 mC cm^{-2} and the one on Sn is 0.065 mC cm^{-2}.

S2.12 Additional data for in situ Raman spectroscopy

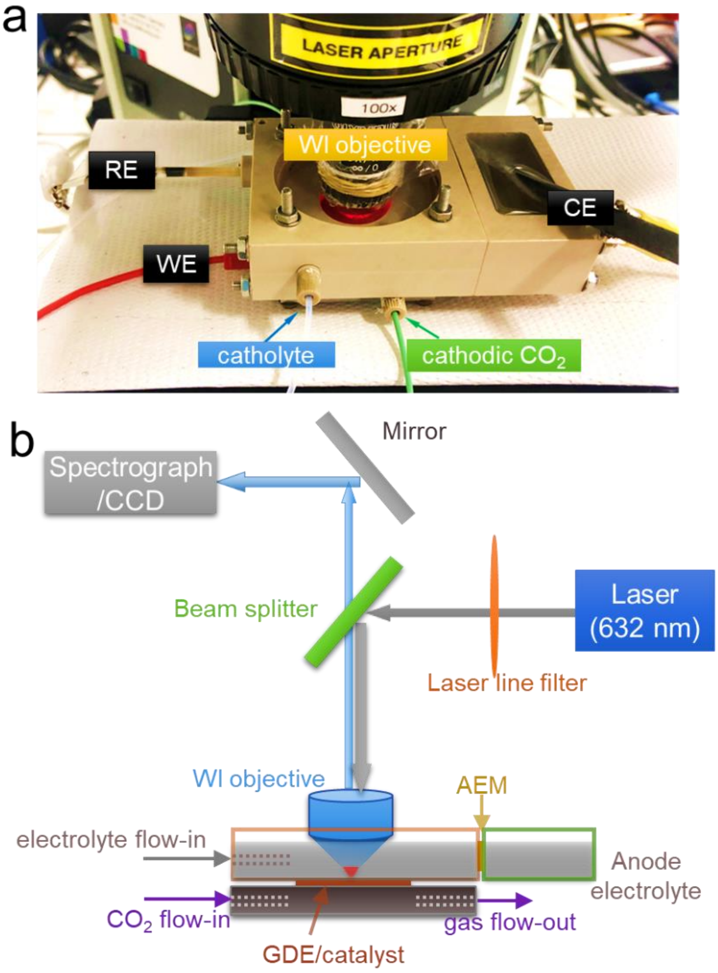

Supplementary Fig. 40. In situ Raman measurement in a flow cell. (**a**) A photograph of the custom-designed flow cell used for in situ Raman measurement during electrochemical CO_2 reduction. (**b**) A schematic diagram of the in situ Raman configuration coupled with a flow cell.

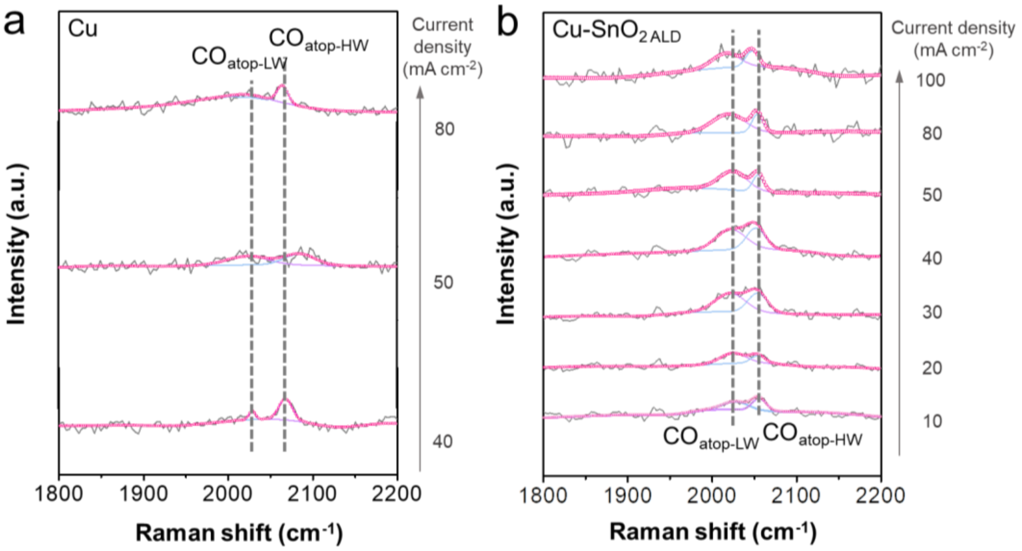

Supplementary Fig. 41. In situ Raman measurement of catalyst in a flow cell. In situ Raman spectra of the C≡O stretch region collected on (**a**) Cu substrate and (**b**) Cu-$SnO_{2\ ALD}$ catalyst. The C≡O stretching feature is decoupled to Raman bands for the linearly adsorbed *CO with low wavenumber ($CO_{atop\text{-}LW}$) and high wavenumber ($CO_{atop\text{-}HW}$).

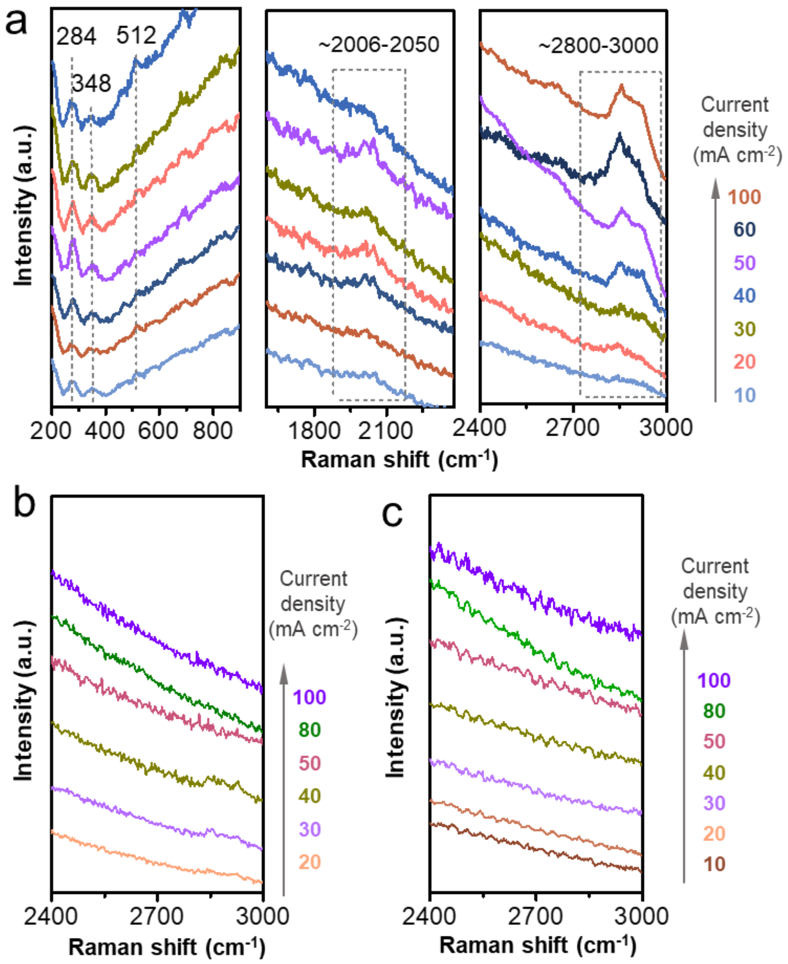

Supplementary Fig. 42. In situ Raman measurement of catalyst in a flow cell. (**a**) In situ Raman spectra of 20-cycle-ALD-Cu-$SnO_{2\ ALD}$ catalyst during CO_2 reduction under different current densities. In situ Raman spectra from 2400 to 3000 cm^{-1} measured on (**b**) Cu and (**c**) 5-cycle-ALD-Cu-$SnO_{2\ ALD}$ catalysts under different current densities.

S2.13 Additional data for solar-driven CO_2 reduction system

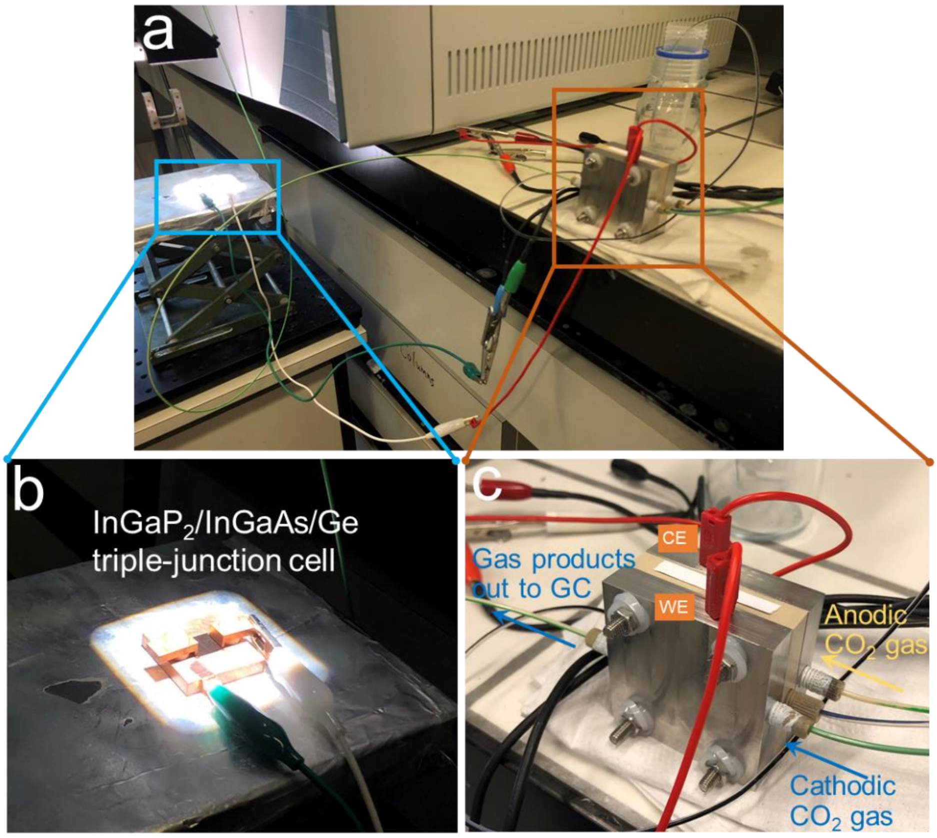

Supplementary Fig. 43. Photographs of solar cell and electrolyzer in solar-driven CO_2 reduction. A photograph of (**a**) the running solar-driven CO_2 reduction system. (**b**) The $InGaP_2$/InGaAs/Ge triple-junction cell used to provide the photocurrent and photovoltage and (**c**) the custom-designed two-electrode electrolyzer. The anion exchange membrane was excluded to minimize the *iR* drop in this reactor and aqueous 2 M KOH was used as the electrolyte to enhance the conductivity.

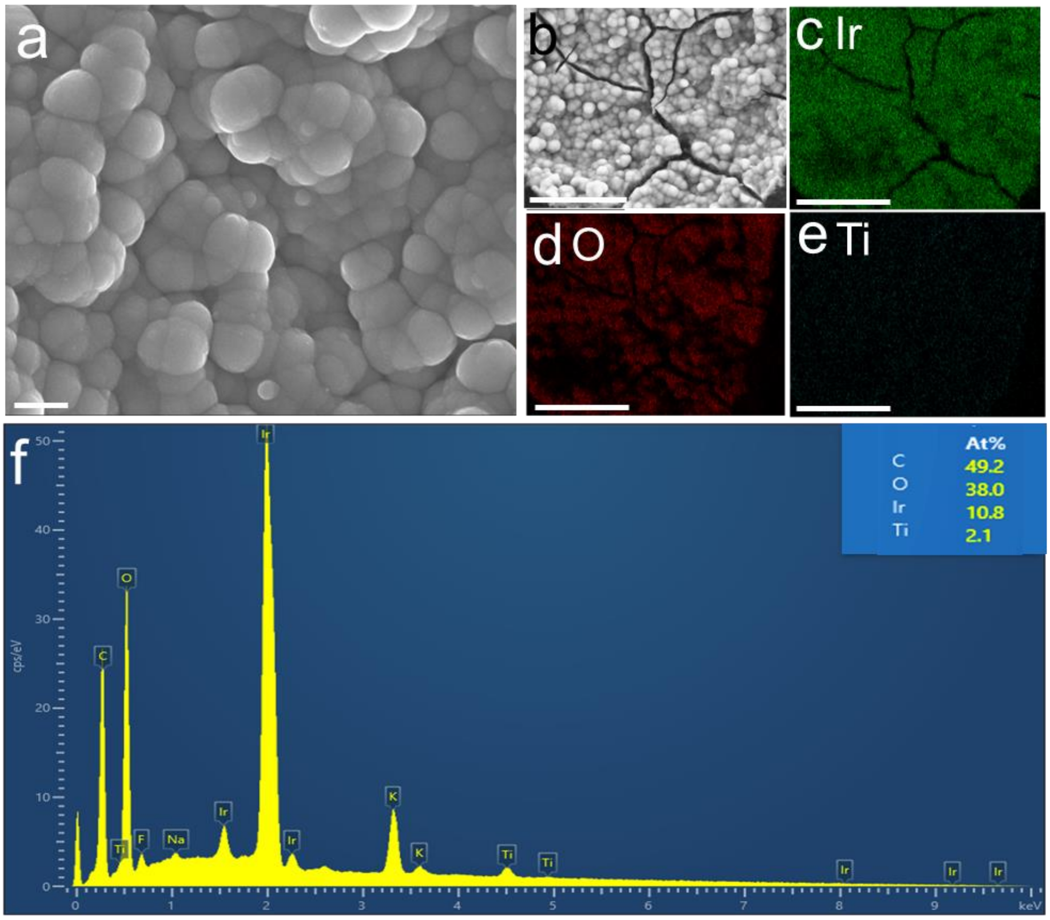

Supplementary Fig. 44. Structural and chemical characterizations of IrO_x catalysts. (**a**) Representative scanning electron micrographs of the electrodeposited IrO_x onto the Ti/GDE substrate. (**b**) Representative secondary electron image of the IrO_x film and the EDX mapping of (**c**) Ir, (**d**) O and (**e**) Ti elements. (**f**) EDX spectrum showing different elements. Scar bars: 500 nm for (a) and 5 μm for (b) to (e).

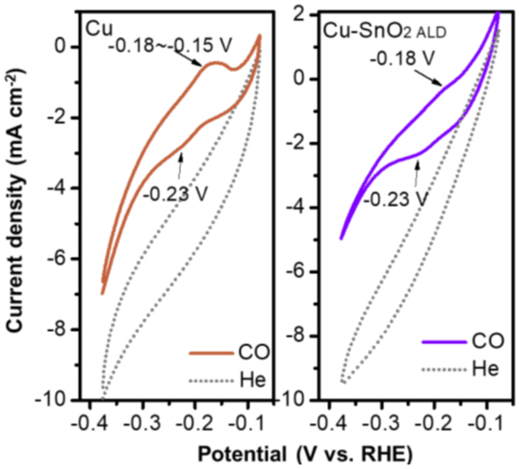

Supplementary Fig. 45. CO adsorption on Cu-SnO_2 ALD catalysts in alkaline electrolyte. Cyclic voltammograms of Cu and Cu-SnO_2 ALD catalysts measured in 2 M KOH electrolyte with He (dash line) and CO (solid line) been flowed at 50 cm^3 min^{-1}, the scan rate is 50 mV s^{-1}. The CO adsorption/stripping features observed in KOH electrolyte are similar with the ones measured in $KHCO_3$, indicating the unchanged catalyst structure in alkaline solution.

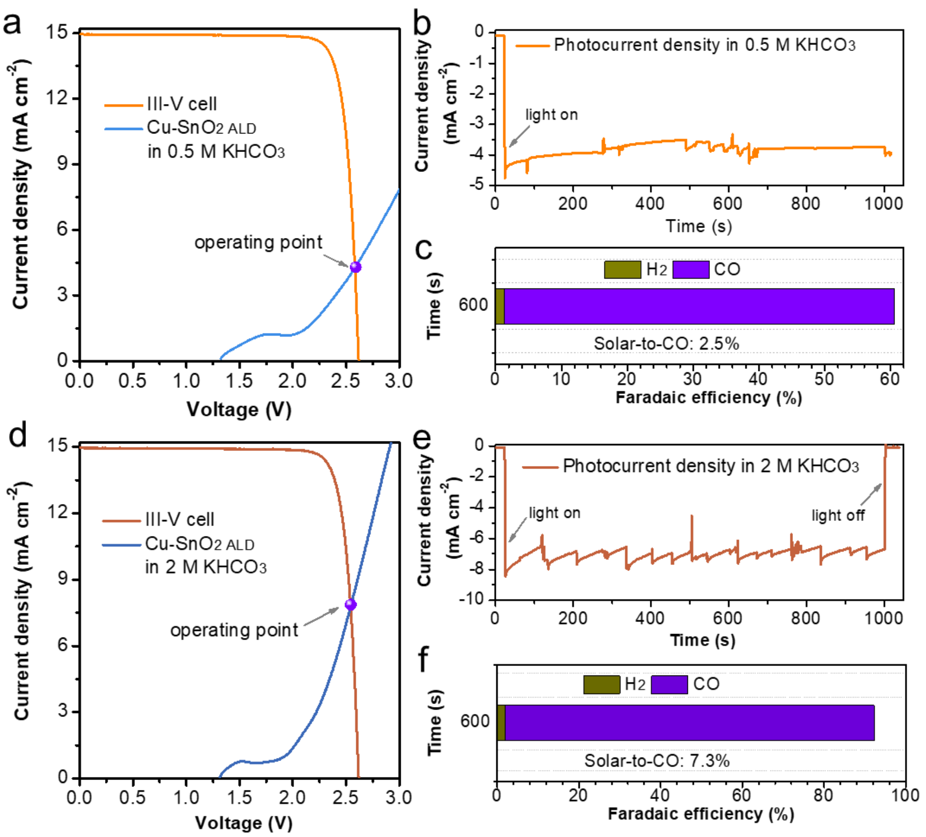

Supplementary Fig. 46. Photosynthesis of CO from solar-driven CO_2 reduction in $KHCO_3$ electrolyte. (**a, d**) Linear weep voltammograms of the solar cell (orange) and electrolysis cell (blue) in 0.5 M and 2 M $KHCO_3$ electrolyte, here the current density of electrolysis is normalized against working area of solar cell. The performance of photovoltaic is measured under simulated standard AM 1.5G with intensity of 100 mW cm^{-2}. (**b, e**) Solar current density and voltage of the unassisted PV-EC system under standard AM 1.5G illumination when 0.5 M and 2 M $KHCO_3$ are used as the electrolyte. (**c, f**) Faradaic efficiency of produced CO and STF conversion efficiency delivered by unassisted PV-EC system under standard AM 1.5G illumination.

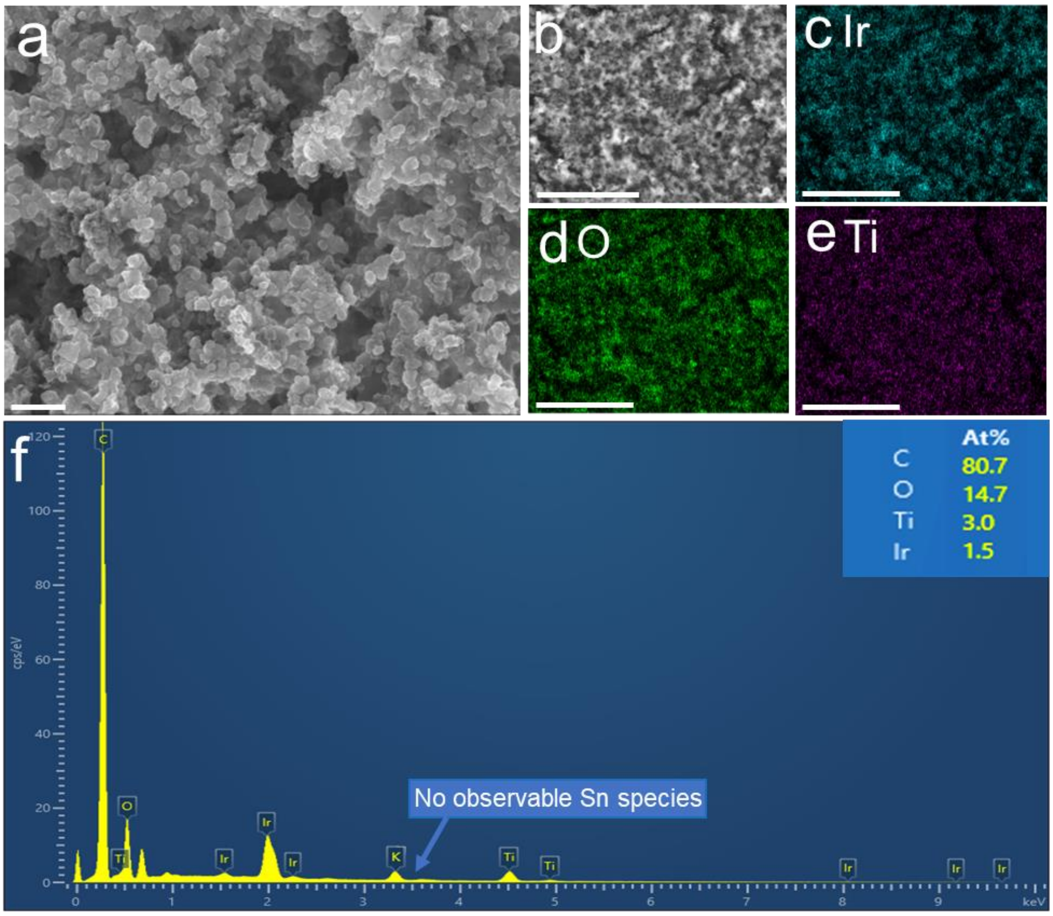

Supplementary Fig. 47. Structural and component characterizations of IrO_x catalyst after solar-driven CO_2 reduction. (**a**) Representative scanning electron micrograph of the IrO_x anode after 3 h solar-driven CO_2 reduction. (**b**) Representative secondary electron image of the IrO_x anode after 3 h solar-driven CO_2 reduction and the EDX mapping of (**c**) Ir, (**d**) O and (**e**) Ti elements. (**f**) EDX spectrum and the table for atomic percentage of various elements. Scar bars: 500 nm for (a) and 5 μm for (b) to (e).

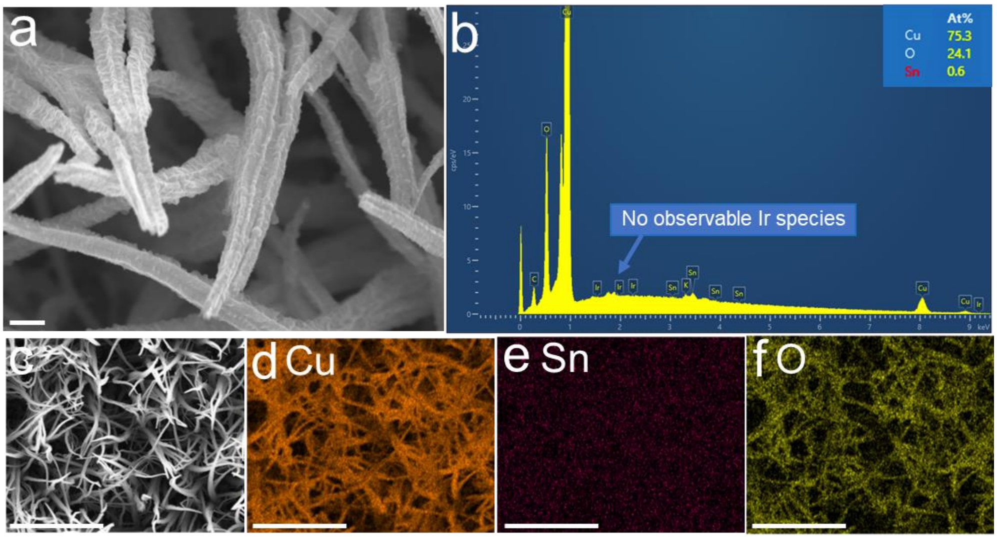

Supplementary Fig. 48. Structural and component characterizations of Cu-SnO_2 ALD catalyst after solar-driven CO_2 reduction. (**a**) Representative scanning electron micrograph of the Cu-SnO_2 ALD after 3 h solar-driven CO_2 reduction. (**b**) EDX spectrum of Cu-SnO_2 ALD after 3 h solar-driven CO_2 reduction. (**c**) Representative secondary electron image of the Cu-SnO_2 ALD after 3 h solar-driven CO_2 reduction and the EDX mapping of (**d**) Cu, (**e**) Sn and (**f**) O elements. Scale bars: 200 nm for (a) and 5 µm for (c) to (f).

S3 Supplementary Tables

Supplementary Table 1. Atomic concentration of elements on the surface of Cu-$SnO_{2\ SER}$ and Cu-$SnO_{2\ ALD}$ catalyst after pre-reduction, as analyzed by XPS.

Sample condition	Samples	C (at %)	O (at %)	K (at %)	Cu (at %)	Sn (at %)
After pre-reduction	Cu-$SnO_{2\ SER}$	29.82	37.33	N.D. [a]	13.80	19.05
	Cu-$SnO_{2\ ALD}$	36.58	33.47	N.D.	26.97	2.98

[a] N.D.: not detected.

Supplementary Table 2. Charge consumed for Pb stripping on Cu active sites from Cu, Cu-$SnO_{2\ SER}$ and SnO_2/Sn catalysts. The values are estimated from cyclic voltammograms shown in Supplementary Fig. 12 and 18, the measurements were carried out in 0.1 M $HClO_4$ + 0.001 M $Pb(OAc)_2$ aqueous electrolyte at a scan rate of 50 mV s^{-1}.

Catalysts condition	Catalysts	Charge consumed for Pb stripping on Cu sites (mC cm^{-2})
After pre-reduction	Cu	3.81 ± 0.46 [a]
	Cu-$SnO_{2\ SER}$	0.54 ± 0.04
	Cu-$SnO_{2\ ALD}$	1.76 ± 0.03
After 50 min-CO_2 reduction	Cu	3.96 ± 0.20
	Cu-$SnO_{2\ SER}$	1.08 ± 0.06
	Cu-$SnO_{2\ ALD}$	2.94 ± 0.02

[a] The charge consumed for Pb stripping correspond to the average value of two to three independent measurements and the error bars are the standard deviations of these measurements. The control curves measured in bare $HClO_4$ shows small anodic current close to zero mA cm^{-2} on all three catalysts (Supplementary Fig. 12), thus we used zero as the background for charge integration.

Supplementary Table 3. The roughness factors of Cu, Cu-$SnO_{2\ SER}$ and Cu-$SnO_{2\ ALD}$ catalysts, estimated from the double layer capacitance shown in Supplementary Fig. 13.

Catalysts	Double layer capacitance ($mF\ cm^{-2}$)	Roughness factor
Cu	2.87 ± 0.3 [a]	94.67
Cu-$SnO_{2\ SER}$	3.16 ± 0.3	105.33
Cu-$SnO_{2\ ALD}$	4.61 ± 0.57	153.67

[a] The capacitance values of the catalysts correspond to the average value of multiple independent measurements and the error bars are the standard deviations of these measurements.

Supplementary Table 4. The dissolved amount of Cu and Sn after CO_2 reduction at -50 mA cm^{-2} for 50 min, as determined by ICP-OES.

Catalyst	Cu	Sn (µg L^{-1})	Sn (µg)
Cu-$SnO_{2\ SER}$	No traces of Cu	40.2	0.54
Cu-$SnO_{2\ ALD}$	No traces of Cu	No traces of Sn	No traces of Sn

Supplementary Table 5. Average faradaic efficiency of the detected products from CO_2 electroreduction on the Cu substrate at different current densities using anion exchange membrane (AEM).

Current density ($mA\ cm^{-2}$)	Faradaic efficiency (%)								
	H_2	CO	CH_4	C_2H_4	$HCOO^-$	CH_3COO^-	C_2H_5OH	C_3H_7OH	Total
-20.0	34.42	23.72	0	5.10	7.01	0.26	0	0	70.50
-30.0	47.58	14.21	0	2.39	7.10	0.08	0	0	71.36
-40.0	39.47	17.87	0	6.63	8.26	0.13	1.30	1.06	74.71
-50.0	28.95	20.08	0	14.01	8.41	0.24	3.47	2.27	77.43
-60.0	23.09	19.74	0	20.35	4.48	0.16	4.28	2.90	74.99
-70.0	16.49	17.39	0	27.59	3.00	0.18	7.70	3.70	76.03
-100.0	13.09	13.03	0	33.66	2.23	0.18	9.46	3.82	75.47
-150.0	20.96	14.52	0.01	38.41	1.99	0.15	9.90	3.08	89.02
-200.0	17.73	9.70	0.10	46.79	1.45	0.21	14.66	4.02	94.64
-250.0	13.93	7.05	0.12	55.42	0.95	0.26	15.69	3.55	96.98

Supplementary Table 6. Average faradaic efficiency of the detected products from CO_2 electroreduction on the Cu substrate at different current densities using cation exchange membrane (CEM).

Current density ($mA\ cm^{-2}$)	Faradaic efficiency (%)								
	H_2	CO	CH_4	C_2H_4	$HCOO^-$	CH_3COO^-	C_2H_5OH	C_3H_7OH	Total
-20.0	62.73	9.93	0	1.27	9.63 [a]	0.14	0.00	0.00	83.70
-30.0	55.14	12.78	0	4.68	15.64	0.25	0.00	0.00	88.49
-40.0	39.29	15.00	0	11.69	14.73	0.35	2.29	1.81	85.15
-50.0	24.49	22.42	0.01	17.67	14.05	0.32	4.01	3.42	86.40
-60.0	18.92	20.03	0.01	21.42	13.63	0.42	6.30	4.94	85.69
-70.0	17.02	17.90	0.04	23.83	11.50	0.44	8.04	5.27	84.04
-100.0	15.02	13.23	0.08	39.15	9.12	0.42	10.28	5.59	92.89

[a] The Faradaic efficiency of $HCOO^-$ obviously increased by replacing AEM by CEM during CO_2 reduction and the total Faradaic efficiency falls into a validated range (84%-93%) at current densities from -20 to -100 $mA\ cm^{-2}$.

Supplementary Table 7. Average partial current density of the detected products from CO_2 reduction on the Cu substrate using anion exchange membrane.

Current density (mA cm^{-2})	Partial current density (mA cm^{-2})							
	H_2	CO	CH_4	C_2H_4	$HCOO^-$	CH_3COO^-	C_2H_5OH	C_3H_7OH
-20.0	6.88	4.74	0	1.02	1.40	0.05	0	0
-30.0	14.27	4.26	0	0.72	2.13	0.02	0	0
-40.0	15.79	7.15	0	2.65	3.30	0.05	0.42	0.28
-50.0	14.47	10.04	0	7.00	4.20	0.12	1.14	1.13
-60.0	13.85	11.84	0	12.21	2.69	0.09	1.74	2.26
-70.0	11.55	12.17	0	19.31	2.10	0.13	2.59	2.46
-100.0	13.09	13.03	0	33.66	2.23	0.18	3.82	3.45
-150.0	31.44	21.78	0.01	57.62	2.98	0.23	14.85	4.63
-200.0	35.46	19.40	0.19	93.57	2.89	0.42	29.31	8.03
-250.0	34.84	17.63	0.30	138.56	2.36	0.66	39.23	8.88

Supplementary Table 8. Average partial current density of the detected products from CO_2 reduction on the Cu substrate using cation exchange membrane.

Current density (mA cm^{-2})	Partial current density (mA cm^{-2})							
	H_2	CO	CH_4	C_2H_4	$HCOO^-$	CH_3COO^-	C_2H_5OH	C_3H_7OH
-20.0	-12.55	-1.99	0.00	-0.25	-1.93	-0.03	0.00	0.00
-30.0	-16.54	-3.83	0.00	-1.41	-4.69	-0.07	0.00	0.00
-40.0	-15.71	-6.00	0.00	-4.68	-5.89	-0.14	-0.92	-0.72
-50.0	-12.25	-11.21	0.00	-8.84	-7.03	-0.16	-2.01	-1.71
-60.0	-11.35	-12.02	-0.01	-12.85	-8.18	-0.25	-3.78	-2.96
-70.0	-11.91	-12.53	-0.02	-16.68	-8.05	-0.31	-5.63	-3.69
-100.0	-15.02	-13.23	-0.08	-39.15	-9.12	-0.42	-10.28	-5.59

Supplementary Table 9. Average faradaic efficiency of the detected products from CO_2 electroreduction on the Cu-$SnO_{2\ SER}$ catalyst at different current densities.

Current density (mA cm^{-2})	Faradaic efficiency (%)							
	H_2	CO	CH_4	C_2H_4	$HCOO^-$	C_2H_5OH	C_3H_7OH	Total
-20.0	0.42	72.79	0	0	1.33	0	0	74.54
-30.0	1.16	92.44	0	0	0.87	0	0	94.47
-40.0	1.15	95.46	0	0	1.51	0	0	98.12
-50.0	0.97	98.21	0	0	1.45	0	0	100.63
-60.0	0.87	96.27	0	0	1.72	0	0	98.86
-70.0	0.81	93.48	0	0	2.22	0	0	96.51
-100.0	0.77	85.05	0	0	4.38	0	0	90.20
-150.0	4.87	72.89	0.06	6.04	3.12	7.01	1.51	95.49
-200.0	3.19	64.39	0.33	5.30	3.86	6.46	0.80	84.34
-250.0	5.17	70.69	1.15	5.05	7.71	7.42	0.62	97.80

Supplementary Table 10. Average partial current density of the detected products from CO_2 reduction on the Cu-$SnO_{2\ SER}$ catalysts.

Current density (mA cm^{-2})	Partial current density (mA cm^{-2})						
	H_2	CO	CH_4	C_2H_4	$HCOO^-$	C_2H_5OH	C_3H_7OH
-20.0	-0.08	-14.56	0	0	-0.27	0	0
-30.0	-0.35	-27.73			-0.26		
-40.0	-0.46	-38.19	0	0	-0.60	0	0
-50.0	-0.49	-49.11	0	0	-0.72	0	0
-60.0	-0.52	-57.76	0	0	-1.03	0	0
-70.0	-0.56	-65.44	0	0	-1.56	0	0
-100.0	-0.77	-85.05	0	0	-4.38	0	0
-150.0	-7.31	-109.33	0.09	-9.05	-4.68	-10.51	-2.26
-200.0	-6.39	-128.79	-0.67	-10.61	-7.72	-12.92	-1.60
-250.0	-12.91	-176.73	-2.87	-12.61	-19.27	-18.55	-1.54

Supplementary Table 11. Average faradaic efficiency of the detected products from CO_2 electroreduction on the Cu-$SnO_{2\ ALD}$ catalyst at different current densities.

Current density (mA cm^{-2})	Faradaic efficiency (%)					
	H_2	CO	C_2H_4	$HCOO^-$	C_2H_5OH	Total
-20.0	0.13	59.15	0	0.91	0	60.19
-30.0	0.20	94.98	0	0.26	0	95.44
-40.0	0.27	98.01	0	0.27	0	98.55
-50.0	0.24	96.17	0	0.32	0	96.73
-60.0	0.20	95.31	0	1.24	0	96.75
-70.0	0.25	93.82	0	0.93	0	95.00
-100.0	0.38	90.16	0	2.00	0	92.54
-150.0	0.90	85.68	0.39	1.76	1.30	88.73
-200.0	0.98	74.72	0.22	3.60	1.42	79.52
-250.0	2.57	82.77	0.40	5.92	1.90	91.66

Supplementary Table 12. Average partial current density of the detected products from CO_2 reduction on the Cu-$SnO_{2\ ALD}$ catalysts.

Current density (mA cm^{-2})	Partial current density (mA cm^{-2})					
	H_2	CO	CH_4	C_2H_4	$HCOO^-$	C_2H_5OH
-20.0	-0.03	-11.83	0	0	-0.18	0
-30.0	-0.06	-28.49	0	0	-0.08	
-40.0	-0.11	-39.21	0	0	-0.11	0
-50.0	-0.12	-48.09	0	0	-0.16	0
-60.0	-0.12	-57.19	0	0	-0.74	0
-70.0	-0.17	-65.67	0	0	-0.65	0
-100.0	-0.38	-90.16	0	0	-2.00	0
-150.0	-1.35	-128.52	0	-0.58	-2.64	-1.95
-200.0	-1.95	-149.44	0.01	-0.44	-7.20	-2.83
-250.0	-6.42	-206.93	0	-0.99	-14.79	-4.76

Supplementary Table 13. The Sn K-post-edge XAS fitting results for the Cu-$SnO_{2\ SER}$ and Cu-$SnO_{2\ ALD}$ catalysts after pre-reduction.

Catalysts	Scatter path	CN	R (Å)	ΔE (eV)	σ^2 (Å^2)
Cu-$SnO_{2\ SER}$	Sn-O	6.6	2.06	6.4	0.00625
	Sn-Sn	4.0	2.69	5.3	0.01266
	Sn-Cu	2.0	3.64	5.3	0.01266
Cu-$SnO_{2\ ALD}$	Sn-O	3.6	2.03	2.2	0.00355
	Sn-Cu	4.6	2.79	4.1	0.00876
	Sn-Sn	3.8	3.58	4.1	0.00876

Supplementary Table 14. The dissolved amount of Cu and Sn after solar-driven CO_2 reduction for ~3 h, as determined by ICP-OES.

Sample	Cu (μg L^{-1})	Cu (μg)	Sn (μg L^{-1})	Sn (μg)
Cu-$SnO_{2\,ALD}$	31.2	0.84	96.2	2.60

Supplementary Table 15. A summary of solar-driven (PV-EC) CO_2 reduction systems. All the systems summarized here are illuminated under standard AM 1.5G unless otherwise stated.

Photovoltaic	Electrolyzer	Cathode	Anode	Catholyte	J_{op} (mA cm^{-2})	Major product/FE	Solar-to-major product	Solar-to-fuel	Ref.
Triple-junction $InGaP_2$/InGaAs/Ge	Flow cell	CuO-SnO_2 $_{ALD}$/GDE	IrO_2	KOH		CO/~100%	19.6%	19.9%	This work
Triple-junction InGaP/GaAs/InGaAs[a]	Flow cell	Ag/GDE	Ni foam	KOH	-14.4	CO/99%	19.1%	19.1%	[6]
GaInP/GaAs tandem	Flow cell	Au_{25}/GDE	NiFe	KOH	-14.1	CO/~100%	~18%	~18%	[7]
a-Si/c-Si[a] heterojunction	Flow cell	Cu/GDE	Se-$(NiCo)S_x/(OH)_x$	KOH	-52.4	C_2H_4/C_2H_5OH	3.9%	n.r.	[8]
GaInP/GaInAs/Ge	H-cell	Ag	Sr_2GaCoO_5	$NaNO_3$	-11.9	CO/87%	14.4%	16.3%	[9]
GaInP/GaInAs/Ge	H-cell	CuO-SnO_2 $_{ALD}$	CuO-SnO_2 $_{ALD}$	$CsHCO_3$	-11.6	CO/81%	13.4%	14.4%	[10]
Perovskite solar cell	H-cell	Au	IrO_2	$NaHCO_3$	-5.8	CO/85%	6.5%	7.0%	[11]
polycrystalline-Si	H-cell	Bi	IrO_2	$KHCO_3$/CsCl	-10	HCOO-/95%	8.5%	n.r.	[12]
Si	H-cell	Cu_2O	IrO_x	$KHCO_3$	-20	C_2H_4/32%	1.5%	4.5%[b]	[13]
Perovskite solar cell	H-cell	CuO	CuO	$CsHCO_3$	-6.0	$C_2H_4+C_2H_6$/40.5%	2.3%	5.5%[b]	[14]
Si tandem	H-cell	Cu/Ag	IrO_2	$CsHCO_3$	-15.1	C_2H_4/~20%	3.5%[b]	5.8%	[15]
Perovskite solar cell	H-cell	CuO_2/Ag	IrO_2	$KHCO_3$	-6.7	C_2H_4	4.2%	7.8%	[16]

[a] a and c represent amorphous and crystalline, respectively.

[b] calculated value based on data shown in the corresponding paper.

n.r. not reported.

S4 Supplementary References

1 *NIST Chemistry WebBOOK, SRD 69*, <https://webbook.nist.gov/cgi/cbook.cgi?Source=1984COX%2FWAG1B&Units=SI&Mask=FFFFFF>

2 Lu, X. *et al.* In Situ Observation of the pH Gradient near the Gas Diffusion Electrode of CO_2 Reduction in Alkaline Electrolyte. *Journal of the American Chemical Society* **142**, 15438-15444 (2020).

3 Kas, R., Kortlever, R., Yılmaz, H., Koper, M. T. M. & Mul, G. Manipulating the hydrocarbon selectivity of copper nanoparticles in CO_2 electroreduction by process conditions. *ChemElectroChem* **2**, 354-358 (2015).

4 Hori, Y., Murata, A. & Takahashi, R. Formation of hydrocarbons in the electrochemical reduction of carbon dioxide at a copper electrode in aqueous solution. *J. Chem. Soc., Faraday Trans. 1* **85**, 2309-2326 (1989).

5 Gu, J., Héroguel, F., Luterbacher, J. & Hu, X. Densely Packed, Ultra Small SnO Nanoparticles for Enhanced Activity and Selectivity in Electrochemical CO_2 Reduction. *Angew. Chem. Int. Ed.* **57**, 2943-2947 (2018).

6 Cheng, W.-H. *et al.* CO_2 Reduction to CO with 19% Efficiency in a Solar-Driven Gas Diffusion Electrode Flow Cell under Outdoor Solar Illumination. *ACS Energy Lett.* **5**, 470-476 (2020).

7 Kim, B. *et al.* Over a 15.9% Solar-to-CO Conversion from Dilute CO_2 Streams Catalyzed by Gold Nanoclusters Exhibiting a High CO_2 Binding Affinity. *ACS Energy Lett.* **5**, 749-757 (2020).

8 Chen, Z. *et al.* Grain-Boundary-Rich Copper for Efficient Solar-Driven Electrochemical CO_2 Reduction to Ethylene and Ethanol. *J. Am. Chem. Soc.* **142**, 6878-6883 (2020).

9 Zhou, L. Q. *et al.* A high-performance oxygen evolution catalyst in neutral-pH for sunlight-driven CO_2 reduction. *Nat. Commun.* **10**, 4081 (2019).

10 Schreier, M. *et al.* Solar conversion of CO_2 to CO using Earth-abundant electrocatalysts prepared by atomic layer modification of CuO. *Nat. Energy* **2**, 17087 (2017).

11 Schreier, M. *et al.* Efficient Photosynthesis of Carbon Monoxide from CO_2 Using Perovskite Photovoltaics. *Nat. Commun.* **6**, 7326 (2015).

12 Piao, G., Yoon, S. H., Han, D. S. & Park, H. Ion-Enhanced Conversion of CO_2 into Formate on Porous Dendritic Bismuth Electrodes with High Efficiency and Durability. *ChemSusChem* **13**, 698-706 (2020).

13 Ren, D., Loo, N. W. X., Gong, L. & Yeo, B. S. Continuous Production of Ethylene from Carbon Dioxide and Water Using Intermittent Sunlight. *ACS Sustainable Chem. Eng.* **5**, 9191-9199 (2017).

14 Huan, T. N. *et al.* Low-cost high-efficiency system for solar-driven conversion of CO_2 to hydrocarbons. *Proc Natl Acad Sci U S A* **116**, 9735-9740 (2019).

15 Gurudayal *et al.* Efficient solar-driven electrochemical CO_2 reduction to hydrocarbons and oxygenates. *Energy Environ. Sci.* **10**, 2222-2230 (2017).

16 Gao, J. *et al.* Selective C–C coupling in carbon dioxide electroreduction via efficient spillover of intermediates as supported by operando Raman spectroscopy. *J. Am. Chem. Soc.* **141**, 18704-18714 (2019).
